# Novel tetrahydroacridine derivatives with iodobenzoic moieties induce G0/G1 cell cycle arrest and apoptosis in A549 non-small lung cancer and HT-29 colorectal cancer cells

**DOI:** 10.1007/s11010-019-03576-x

**Published:** 2019-07-16

**Authors:** Małgorzata Girek, Karol Kłosiński, Bartłomiej Grobelski, Stefania Pizzimenti, Marie Angele Cucci, Martina Daga, Giuseppina Barrera, Zbigniew Pasieka, Kamila Czarnecka, Paweł Szymański

**Affiliations:** 1 Department of Pharmaceutical Chemistry, Drug Analyses and Radiopharmacy, Faculty of Pharmacy, Medical University of Lodz, Muszynskiego 1, 90-151 Lodz, Poland; 2 Department of Experimental Surgery, Faculty of Medicine, Medical University of Lodz, Pabianicka 62, 93-513 Lodz, Poland; 3 Animal House, Faculty of Pharmacy, Medical University of Lodz, Muszynskiego 1, 90-151 Lodz, Poland; 4 Department of Clinical and Biological Sciences, School of Medicine, University of Turin, Corso Raffaello 30, 10125 Turin, Italy

**Keywords:** A549, HT-29, Cytotoxicity, Apoptosis, Cell cycle arrest, PARP-1

## Abstract

A series of nine tetrahydroacridine derivatives with iodobenzoic moiety were synthesized and evaluated for their cytotoxic activity against cancer cell lines—A549 (human lung adenocarcinoma), HT-29 (human colorectal adenocarcinoma) and somatic cell line—EA.hy926 (human umbilical vein cell line). All compounds displayed high cytotoxicity activity against A549 (IC_50_ 59.12–14.87 µM) and HT-29 (IC_50_ 17.32–5.90 µM) cell lines, higher than control agents—etoposide and 5-fluorouracil. Structure–activity relationship showed that the position of iodine in the substituent in the *para* position and longer linker most strongly enhanced the cytotoxic effect. Among derivatives, **1i** turned out to be the most cytotoxic and displayed IC_50_ values of 14.87 µM against A549 and 5.90 µM against HT-29 cell lines. In hyaluronidase inhibition assay, all compounds presented anti-inflammatory activity, however, slightly lower than reference compound. ADMET prediction showed that almost all compounds had good pharmacokinetic profiles. **1b**, **1c** and **1f** compounds turned out to act against chemoresistance in cisplatin-resistant 253J B-V cells. Compounds intercalated into DNA and inhibited cell cycle in G0/G1 phase—the strongest inhibition was observed for **1i** in A549 and **1c** in HT-29. Among compounds, the highest apoptotic effect in both cell lines was observed after treatment with **1i**. Compounds caused DNA damage and H2AX phosphorylation, which was detected in A549 and HT-29 cells. All research confirmed anticancer properties of novel tetrahydroacridine derivatives and explained a few pathways of their mechanism of cytotoxic action.

## Introduction

It was estimated that there would be 18.1 million new cases and 9.6 million cancer deaths worldwide in 2018. Lung cancer affects 2.1 million people (11.6% of total cases) and causes death of 1.76 million (18.4% of total cancer deaths). These numbers give lung cancer number one among all cases, and it is the leading cause of cancer deaths worldwide. Colorectal cancer (CRC) would be responsible for 1.8 million new cases (10.3% of total incidents) and for 0.88 million (9.2% of total cancer deaths) of mortality due to the cancer. It gives colon cancer third place among all new cases and second cause of death. New cancer cases and mortality are growing worldwide [1]. Chemotherapy has many side effects such as pain, vomiting, diarrhoea, stomatitis, vascular and neuronal damage. Moreover, anticancer drugs possess poor bioavailability, fast renal clearance and can induce resistance [2–4]. Among drugs using to treat colon cancer, 5-fluorouracil (5-FU) is the basic one. It inhibits DNA replication and further blocks the growth of cancer cells [2, 5]. Etoposide is an anticancer drug which is the topoisomerase II inhibitor [6, 7]. Etoposide is used to treat lung cancer, testicular cancer, lymphoma or glioblastoma multiform [8]. Anticancer agents, such as etoposide, bleomycin and doxorubicin, damage the vascular endothelium. Because of these treatments, which can lead to necrosis or apoptosis, the endothelium loses its integrity and preservation of many functions [9].

Acridines have a long story of being therapeutic compounds. They are known as antibacterial, antiviral and anticancer agents [10]. Nowadays, the most common acridine derivative is amsacrine, which is used as antineoplastic agents in the treatment of leukaemia. Moreover, researchers around the world have studied novel acridine derivatives against various cancers [11–17]. The mechanism of action of acridine derivatives involves intercalation into DNA in the space between two base pairs, due to the planar polycyclic aromatic structure. Intercalation results in unwinding of double helix and lengthening of the DNA strand. These changes can cause retardation or inhibition of transcription and replication, which is a good feature in cancer treatment, but might result in mutagenic activity in somatic cells. [18].

Oxidative stress has a role in cancer development. Cancer itself increases oxidative stress. In cancer cells, oxidative stress is associated with mitochondrial dysfunction, increased levels of reactive oxygen species (ROS) and higher metabolic activity. Moreover, oxidative stress in cancer cells stimulates cellular proliferation, promotes mutations and alternates cell’s sensitivity to anticancer agents. Some chemotherapeutic agents can generate ROS in patients’ cells during treatment [19]. ROS is important for the induction of apoptosis in many cancer cells [20], and can cause DNA damage [21]. Antioxidants inhibit ROS generation and some might say that by this way, use of antioxidants can prevent apoptosis in cancer cells. There is a different opinion of the use of antioxidants during anticancer treatment [22, 23], because anticancer agents generate ROS and antioxidants may prevent cancer cells by inhibiting ROS generation. Therefore, it is believed that simultaneously use of antioxidants should be avoided during cancer treatment [24]. However, except for three interferences (tangeretin with tamoxifen, NAC with doxorubicin and beta-carotene with 5-fluorouracil), many data show the increase in effectiveness as well as decrease of side effects of chemotherapeutic drugs when administrated with antioxidants [25]. Even though many anticancer drugs induce ROS, their anticancer effects do not only depend on free radicals [26–28]. Although clinical studies on the effect of antioxidants in cancer treatment are limited, experimental studies show that antioxidant vitamins could induce apoptosis in cancer cells, prevent angiogenesis and metastatic spread. This could have an impact on potential role of antioxidants as adjuvants in anticancer therapy [29].

Studies on biodistribution of tetrahydroacridine derivatives with similar structure showed that these compounds accumulate mainly in the rat’s lungs and intestines [30]. A series of novel tetrahydroacridine and cyclopentaquinoline derivatives with fluorobenzoic acid moieties were synthesized and evaluated for their anticancer properties by our research team previously [13, 31]. Results became the basis for further researches in this direction. In current study, we evaluated next group of tetrahydroacridine derivatives—the ones with iodobenzoic moieties. Moreover, benzoic acid derivatives possess antioxidant activities [32]. Due to the iodobenzoic moiety, we checked if our compound with iodobenzoic moiety has its role in antioxidant action. The influence of novel kind of moieties on anticancer activities of tetrahydroacridine derivatives was investigated. The synthesis of novel tetrahydroacridine compounds with iodobenzoic acid moiety was described previously [33]. The aim of this study was to determine the cytotoxic activity of 9 novel tetrahydroacridine derivatives on A549, HT-29 cell lines, to investigate their biological properties and molecular mechanism of action.

## Materials and methods

### Synthesis of tetrahydroacridine derivatives

**Scheme 1:**
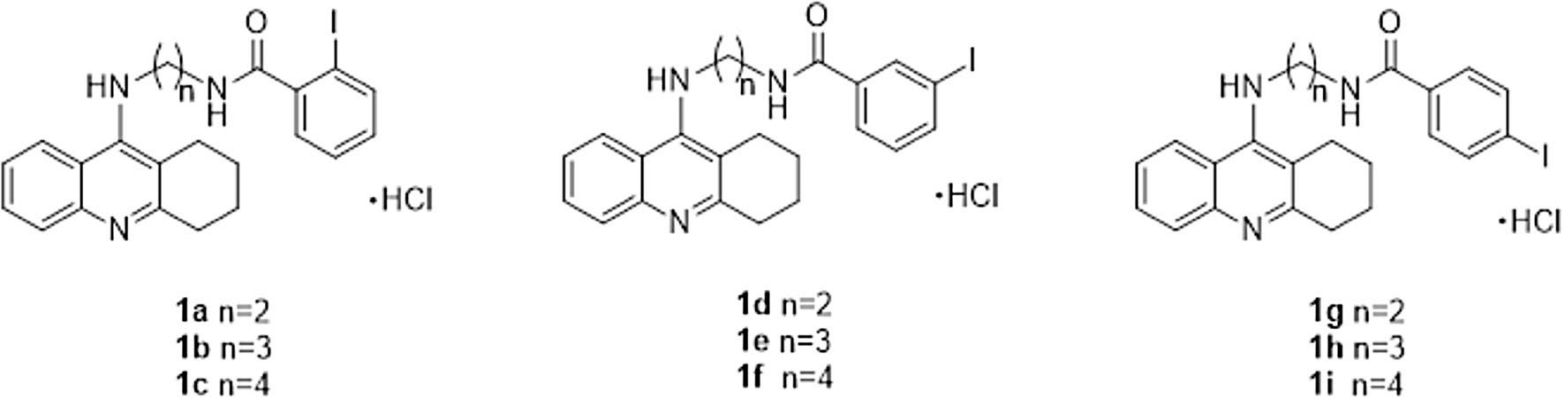
Tetrahydroacridine derivatives **1a**–**1i**

The synthesis of novel tetrahydroacridine derivatives with iodobenzoic moieties (Scheme 1) was described previously in publication by Skibiński et al. [33]. At the beginning, the 2-chloro-4,6-dimethyl-1,3,5-triazine (CDMT) in THF (10 mL), 2-iodobenzoic acid, 3-iodobenzoic acid or 4-iodobenzoic acid and dropwise *N*-methylmorpholine were mixed together at temperature − 10 °C. Mixture was stirred for 2 h. Then amines (0.16–0.36 g) dissolved in THF (5 mL),were added at − 10 °C to the mixture. Stirring was continued at room temperature for 24 h. Obtained compounds were purified by flash chromatography. In order to get hydrochloride salts, compounds were dissolved in methanol (0.5 mL) and then HCl/ether (10 mL) was added. Firstly, mixture was stirred at room temperature for 30 min and then was stirred overnight to form solid. The compounds were isolated by filtration and dried.

### ADMET analysis

Absorption, distribution, metabolism, elimination and toxicity (ADMET) analysis describes predictable disposal of novel compounds within the body. During the development of new drugs, it is crucial to find out, whether compounds violate Lipinski’s rule of five, or cause genotoxicity and cardiotoxicity. Lipinski’s rule of five is used to evaluate pharmacological and biological activities which determine likeness of being orally active drugs. ADMET predictions were calculated using ACD/Percepta 14.0.0 [34].

### Cell culture

A549 cell line (lung carcinoma from human) (European Collection of Cell Culture) and HT-29 cell line (colorectal adenocarcinoma from human) (American Type Culture Collection) were chosen to evaluate the cytotoxicity of novel compounds. A549 cells were grown in Dulbecco’s Modified Eagle’s Medium (PAN-Biotech) containing 10% Fetal Bovine Serum (FBS) (Sigma Aldrich), 2 mM Glutamine (Sigma Aldrich) and 100 units/mL penicillin and 100 mg/mL streptomycin (Sigma Aldrich).

HT-29 cells were grown in McCoy’s Medium (Biological Industries) containing 10% FBS (Sigma Aldrich), 2 mM Glutamine (Sigma Aldrich) and 100 units/mL penicillin and 100 mg/mL streptomycin (Sigma Aldrich).

The EA.hy926 cells (the human umbilical vein, somatic cell hybrid) (American Type Culture Collection) are immortalized human umbilical vein endothelial cells. They have morphological, phenotypic and functional characteristics of human endothelial cells, without the limited lifespan. This cell line has been considered as a good model for anticancer drug screening [35] and was chosen to evaluate the endothelial cytotoxicity of novel compounds. Cells were grown in Dulbecco’s Modified Eagle’s Medium (PAN-Biotech) containing 10% FBS (Sigma Aldrich), 2 mM Glutamine (Sigma Aldrich) and 100 units/mL penicillin and 100 mg/mL streptomycin (Biological Industries).

253J B-V (human bladder cancer) (kindly provided by Dr Colin Dinney—MD Anderson Cancer Center) and 253J B-V cisplatin-resistant cells (253J B-V Cr) were chosen to evaluate if novel compounds were cytotoxic to sensitive and chemoresistant cells. Cells were grown in RPMI 1640 medium (EuroClone), supplementing with 10% FBS (EuroClone), 100 units/mL penicillin and 100 mg/mL streptomycin (EuroClone).

Induction of Cisplatin resistance was induced as previously described—resistance to cisplatin was induced by adding 253J B-V cells to various concentrations of cisplatin (0.5; 0.8; 1; 1.3; 1.5 μg/mL). Each concentration was tested for at least 6 weeks. After this time, cells stayed for at least 1 month without addition of the drug. When they started to proliferate again, cisplatin concentration was increased to the highest dose. It took a year to induce resistance to the highest cisplatin concentration [36].

The SH-SY5Y (human neuroblastoma) (European Collection of Cell Culture) was chosen to evaluate the protection from oxidative stress. Cells were grown in Ham’s F12:EMEM (1:1) (Sigma Aldrich) containing 2 mM Glutamine (Sigma Aldrich), 1% Non-Essential Amino Acids (Biological Industries), 15% FBS (Biowest) and 100 units/mL penicillin and 100 mg/mL streptomycin (Biological Industries).

All cell lines grown in an incubator at 37 °C with 5% CO_2_ to 80% confluence before the initiation of the assay.

### MTT cytotoxicity assay

The MTT assay was used to evaluate cell metabolic activity. The test principle is based on the determination of cell viability by measuring the metabolic activity in mitochondria. Enzymatically active mitochondria reduce the water-soluble MTT (3-(4,5-dimethylthiazol-2-yl)-2,5 diphenyltetrazoliumbromide) to blue–violet insoluble formazan. The number of viability cells is proportional to the colour intensity determined by photometric measurements (an analytical wavelength 570 nm) after dissolving the formazan in Dimethylsulfoxide (DMSO) (Serva) and Sorensen’s glycine buffer. MTT (Sigma Aldrich) was dissolved in Phosphate Buffered Saline (PBS) (PAN-Biotech) at a concentration of 0.5 mg/mL. Sorensen’s glycine buffer is made of 0.1 M glycine and 0.1 M NaCl, at pH 10.5. Studied compounds were dissolved in DMSO in order to receive stock solutions.

For the assay, cells were seeded into 96-well plates at density 10^4^ cells per well and cultured for 24 h at 37 °C and 5% CO_2_. Then medium was removed, and cells were exposed to the 100 µL of the compound solutions over a range of concentrations (final DMSO concentration was below 0.2%) or nothing but culture medium (blank control). Pure DMSO was used as a positive control. After 24 h of incubation with the compound solutions, medium was removed and 50 µL of the MTT solution was added to each well and incubated in the dark for additional 2 h at 37 °C. Then the MTT solution was carefully removed and 100 µL of DMSO was added. Plates were incubated for 10 min at room temperature. Finally, 5 µL of Sorensen Buffer was added to each well. Plate was swayed, and the absorbance was measured by using microplate reader (Synergy H1, BioTek, Winooski, VT, USA) at a wavelength of 570 nm. The cell viability was expressed as a percentage of the control values (blank). The experiments were done in triplicate [37].

### Colony forming assay

The A549 or HT-29 cells were seeded in 6-well plates (1000 cells/well). After 24 h, solutions of the test compounds at the concentrations of 2xIC_50_, IC_50_, _1/2_IC_50_ were added. The cells were incubated for a further 24 h, after which the culture medium was changed. The cells were then cultured until clear colonies were formed. For the staining of cells, a 5% Giemsa solution (Sigma Aldrich) was prepared. After the incubation, the culture medium was gently removed from each well. The plates were placed on ice. Each well was rinsed twice with cold PBS (1 mL). The cells were fixed with cold methanol (1 mL) for 20 min. The methanol (Serva) was removed, and then the fixed colonies were stained with 5% Giemsa for 10 min at room temperature. After the incubation, the Giemsa solution was removed. The colonies were rinsed with distilled water to remove the excess of the dye. Plates were left to dry and then colonies in each well were photographed under microscope and calculated. The colony is defined to consist of at least 50 cells. Each experiment was repeated three times in triplicate. Mean number of colony numbers in control wells were expressed as 100%. Number of colonies in experimental wells were expressed as percent of control number. Results were presented as the mean ± SD of three separate experiments [38].

### Cell cycle analysis

A549 or HT-29 cells were seeded on 6-well plates at the density of 2.5 × 10^5^ cells/well and incubated for 24 h. Then, compounds were added into the wells and cells were further incubated for 24 h. After incubation, monolayer and supernatant cells were harvested and washed in PBS. Then, cells were centrifuged (monolayer—1200 rpm for 10 min, supernatant 3500 rpm for 10 min) and fixed in cold 70% ethanol. Pellets were kept at 4 °C for at least 2 h. Prior to analysis, cells were centrifuged and re-suspended in RNAsi (type 1-A; Sigma Aldrich)—PBS working solution (200 µg/mL). Samples were incubated at 37 °C for 10 min. At the end, 3 µL of 2 mg/mL propidium iodide (PI) (Sigma Aldrich) solution was added to each sample. Samples were kept in the dark prior to analysis. Analysis was performed on BD Accuri C6 Flow Cytometry (Becton–Dickinson). Etoposide (Calbiochem) was used as a reference drug. Experiments were done in triplicates [39].

### Apoptosis assay by annexin-V/PI double staining

A549 or HT-29 cells were seeded on 6-well plates at the density of 2 × 10^5^ cells/well and incubated for 24 h. Compounds were added to the cells and further incubated for 24 h. After treatment, monolayer and supernatant cells were harvested, washed with PBS and centrifuged (monolayer at 1200 rpm for 10 min, supernatant at 3500 rpm for 10 min). Then, cells were re-suspended in binding buffer and stained with 2 µL of Annexin-V and 2 µk of PI (ANNEXIN-V—FITC Apoptosis Detection Kit, Immunostep) in the dark for 10 min at room temperature. Cells were analysed by BD Accuri C6 Flow Cytometry (Becton–Dickinson). Etoposide (Calbiochem) was used as a reference drug. Experiments were done in triplicates [39].

### Hyaluronidase inhibition assay

All compounds were tested in a hyaluronidase inhibition test in order to determine their anti-inflammatory properties. The hyaluronidase inhibition study was carried out by turbidimetric method modified to the 96-well plates and previously described by Michel et al. [40]. Compound solutions were prepared freshly before the assay. The protocol started by adding 20 µL of the compound solution in monosodium phosphate buffer (pH 7.0) and 40 µL of hyaluronidase solution (22.55 U/mL, hyaluronidase from bovine testes Type I-S, Sigma Aldrich) to the wells of 96-well microtiter plates. The solutions were kept in the dark for 10 min at the temperature 37 °C. After incubation, 40 µL of hyaluronic acid solution (0.03%, Sigma Aldrich) in monosodium phosphate buffer (pH 5.35) was added to the wells. Then the mixture was incubated in the dark for 45 min at 37 °C. Finally, 300 µL of bovine serum albumin (0.1%, Serva) in sodium acetate buffer (pH 3.75) was added to the wells. The final incubation was carried out at the room temperature for 10 min. The changes in turbidity were measured at 600 nm by a microplate reader (BioTek, Winooski, VT, USA). The assay was run in three experiments in triplicate in order to calculate IC_50_ values. Heparin (WZF, Polfa Warsaw) was a positive control. The inhibitory activity of the tested compounds was calculated as inhibition percentage (% inhibition) of hyaluronidase according to the equation:

$$\%\, {\text{Inhibition}} = 100 \times \left( {1 - \left( {\frac{{A_{\text{HA}} - A_{\text{AN}} }}{{A_{\text{HA}} - A_{\text{HYAL}} }}} \right)} \right),$$

where *A*_HA_—absorbance of solution without the enzyme (positive control), *A*_HYAL_—absorbance of solution without the tested compound (negative control), *A*_AN_—absorbance of solution with the tested compound.

### Cytoprotection against oxidative stress

The cytoprotective properties of novel compounds against oxidative stress were measured in three experiments. In the first experiment, hydrogen peroxide (H_2_O_2_) was used to generate exogenous free radicals. Cells were incubated with compounds in the concentrations of IC_50_ (and lower) for 24 h before the addition of H_2_O_2_ (100 µM). Then, H_2_O_2_ was added and the incubation in the presence of compounds lasted 24 h. In the second and third experiment, combination of rotenone (30 µM) (Sigma Aldrich) with oligomycin A (10 µM) (Sigma Aldrich) was used to induce mitochondrial reactive oxygen species (ROS). In the second experiment (pre-incubation), cells were incubated with compounds for 24 h before the addition of rotenone with oligomycin A. Then, the toxic agents were added and the cell incubation in the presence of compounds was maintained for additional 24 h. In the third experiment (co-incubation), compounds and the combination of rotenone with oligomycin A were added at the same time and then incubated for 24 h. In each experiment, the cell death was evaluated by the MTT assay. Data were shown as the percentage of the reduction of MTT in regard to non-incubated cells. Trolox (the moiety of antioxidant vitamin E) (Sigma Aldrich) was used as a positive control. Each experiment was repeated three times in quadruplicates. Data in the tables were showed as the percentage of protection—for example loss of cell viability by 25% in the drug-treatment sample, means protection of 50% [41, 42].

### Reactive oxygen species assay

A549 or HT-29 cells were seeded in 6-well plates at the density of 2.5 × 10^5^ cells/well and incubated for 24 h. After incubation, compounds were added into the wells and further incubated for next 24 h. H_2_O_2_ (1 mM) was added at 22nd h of incubation with compounds. H_2_O_2_ was used as a positive control. 30 min before the analysis, DCFH-DA (20 µM) was added to the wells. After incubation, cells were harvested and washed in PBS. Then, cells were centrifuged at 1500 rpm for 10 min. Medium was removed and cells were suspended in PBS. Analysis was performed on BD Accuri C6 Flow Cytometry (Becton–Dickinson) [39].

### DNA unwinding assay

0.3 µg of supercoiled pUC19 DNA (0.5 µg/µL) (Thermofisher) was mixed with 10 × assay buffer (100 mM Tris–HCl (pH 7.9), 10 mM EDTA, 1.5 M NaCl, 1% BSA, 1 mM spermidine, 50% glycerol) (TopoGen), 2 µL of solutions of compounds in chosen concentrations (except of two controls) and water. Human Topoisomerase I (TopoI) (4U) (TopoGen) was added to the all samples, except one of the controls. Dilution buffer (20 mM NaH_2_PO_4_ (pH 7.4), 300 mM NaCl) (TopoGen) was added to the control. Amsacrine (Cayman) and ethidium bromide (EB) (Serva) were used as reference compounds. Final volume per sample was 20 µL. All reactions were carried out in DNAse-free microcentrifuge tubes. Mixtures were gently mixed and incubated in the water bath for 30 min in 37 °C. Reaction was terminated by adding 10% SDS (final concentration 1%) and 0.6 mg/mL solution of proteinase K (Serva) (final concentration 0.05 mg/mL), which was pre-warmed at 37 °C for 10 min. 6 × Loading Dye (Thermofisher) was added to each sample. Samples were analysed in 0.8% agarose gel in TAE (Tris–acetate-EDTA, pH 8.30) buffer at 4 V/cm for 4 h at the 20 °C. After analysis, gel was stained in the dark with EB solution (0.05 µg/mL) for 15 min, de-stained in water for 5 min and visualized with UV [43].

### Protein extraction and Western Blotting analysis

A549 or HT-29 cells were seeded in 6-well plates at the density of 2.5 × 10^5^ cells/well and incubated for 24 h. Then, cells were treated with chosen compounds. After 24 h of incubation, monolayer and supernatant were harvested and washed in cold PBS. Cells were centrifuged—monolayer at 1200 rpm for 10 min, supernatant 3500 rpm for 10 min. Pellets were dissolved in RIPA Lysis and Extraction buffer (Bio-Rad) containing Phosphatase Inhibitor Cocktail 3 (Sigma Aldrich) and Protease Inhibitor Cocktail (Sigma Aldrich). Samples were incubated in ice for 30 min and mixed every 10 min. Then, samples were centrifuged at 12,000 rpm for 25 min. Protein concentration in supernatants was measured in triplicate using a Protein Assay (Bio-Rad). Proteins were separated by SDS–polyacrylamide gel electrophoresis. Separating and stacking gels were made of different ratios of water, Tris–HCl pH 6.8 and pH 8.8, 10% SDS (Sigma Aldrich), Acrylamide/bis-acrylamide (Sigma Aldrich), 10% APS (Sigma Aldrich), TEMED (Sigma Aldrich). 7.5% gels were prepared for analysis PARP-1, whereas 12% gels prepared for γ-H2AX. After separation on SDS–polyacrylamide gels, proteins were electroblotted on nitrocellulose membrane in Transfer Buffer (Glycine, Tris, Methanol). Membranes were washed in TBS/0.1% or 0.05% Tween20 solution and blocked in Tris-buffered saline with 5% of non-fated dried milk for 1 h. Membranes were then incubated overnight with primary antibodies—anti-PARP-1 (Cell Signaling Technology), anti-γ-H2A.X (Cell Signaling Technology), anti-β-actin (Cell Signaling Technology) in cold room (6 °C). After incubation, membranes well washed with TBS/Tween solution and blocked with and horseradish peroxidase-conjugated secondary antibodies (Bio-Rad) for 1 h in cold room. Protein bands were visualized using ECL™ Prime Western Blotting Detection Reagents (Sigma Aldrich) Healthcare and Carestream Kodak autoradiography GBX fixer/replenisher (Sigma Aldrich) with Developer and Replenisher (Carestream) in the dark room. Results were obtained on Autoradiography Film (Santa Cruz Biotechnology). Densitometric analysis was performed by using a software program (ImageJ). Results were standardized using the signal obtained from β-actin [44].

### Statistical analysis

Obtained values were represented as mean ± standard deviation (SD) from experiments. One-way ANOVA with post hoc analysis was made using the Stat-Soft STATISTICA v.13.1 software.

## Results

### ADMET Analysis

ADMET analysis informs researchers about safety and probability of success in the development of novel compounds. The values for every compound (**1a**–**1i**) are presented in Table 1. Following the Rule of five, molecular weight was lower than 500, number of hydrogen bond donors (expressed as the sum of OHs and NHs) were lower than 5, number of hydrogen bond acceptors (expressed as the sum of Ns and Os) were lower than 10, number of rotatable bonds were lower than 10 and an octanol–water partition coefficient log *P* not greater than 5. Almost all compounds did not violate Lipinski’s Rule of five, however, **1e**–**1i** had log *P* slightly higher than 5. Other parameters were not violated. The Rule of five got its name from the cut-offs of the parameters which are close to 5 or multiple of 5 [45–48]. Drugs, which violate the Rule, have poor absorption or permeation after oral intake. However, some drugs lie outside the parameters of the Rule, for example antibiotics, antifungals, vitamins and cardiac glycosides. It may be due to their structural features which allow drugs to act as substrates for transporters.

**Table 1:** ADMET prediction values for novel compounds **1a–1i**

	**1a**	**1b**	**1c**	**1d**	**1e**	**1f**	**1g**	**1h**	**1i**	Tacrine
Molecular weight	471.33	485.36	499.39	471.33	485.36	499.39	471.33	485.36	499.39	200.28
No. of H-bond donors	2.00	2.00	2.00	2.00	2.00	2.00	2.00	2.00	2.00	2.00
No. of H-bond acceptor	4.00	4.00	4.00	4.00	4.00	4.00	4.00	4.00	4.00	2.00
No. of rotatable bonds	5.00	6.00	7.00	5.00	6.00	7.00	5.00	6.00	7.00	0.00
Log *P*	4.79	5.02	5.01	5	5.23	5.21	5.42	5.67	5.66	2.60
TPSA (Å^2^)	54.02	54.02	54.02	54.02	54.02	54.02	54.02	54.02	54.02	38.38
Log BB	0.48	0.47	0.55	0.55	0.53	0.59	0.55	0.52	0.58	0.21
Probability of positive Ames test	0.72	0.61	0.68	0.72	0.61	0.68	0.72	0.61	0.68	0.77
*P*-gp inhibitor probability	0.56	0.68	0.60	0.56	0.68	0.60	0.56	0.68	0.60	0.16
*P*-gp substrate probability	0.43	0.54	0.68	0.43	0.54	0.68	0.43	0.54	0.68	0.12
hERG Inhibitor (Ki < 10 µM) probability	0.56	0.51	0.56	0.56	0.51	0.56	0.56	0.51	0.56	0.10
%PPB	96.67	97.08	97.31	96.67	97.08	97.31	96.67	97.08	97.31	76.27
Log *K*_a_^HSA^	5.15	5.15	4.90	5.15	5.15	4.90	5.15	5.15	4.90	3.73
*V*_d_ (L/kg)	8.06	8.40	9.50	11.06	11.16	12.63	11.39	11.49	13.01	5.06

Parameters: Log *P*—an octanol–water partition coefficient; TPSA (Å^2^)—topological polar surface; Log BB—Blood–Brain Barrier Penetration log([Brain]/[Blood])); *P*-gp—*P*-glycoprotein; hERG—the human Ether-a-go-go-Related Gene; %PPB—plasma protein binding; Log *K*_a_^HSA^—the human serum albumin affinity constant; Vd - the distribution of compounds between plasma and body tissue

The blood–brain barrier (BBB) separates blood circulation and cerebrospinal fluid in the central nervous system. By prediction BBB penetration, researchers may find out whether compounds pass across the barriers. All of novel compounds have log BBB not lower than − 1 and higher than 0.3 (0.48–0.59), which show good brain penetrations profiles. The iodine in *para* position and longer carbon chain improved BBB permeability [47]. TPSA is the topological polar surface area which derived only from polar fragments with nitrogen and oxygen. Low topological polar surface area (< 75 Å^2^) may indicate an increase in in vivo toxicity. Moreover, the relationship between clog P and TPSA is observed. Compounds with values of clog *P* < 3 and TPSA > 75 Å^2^ are considered to be non-toxic [49, 50]. All of our novel compounds have TPSA slightly below 75 Å^2^ (54.02 Å^2^), which may indicate some toxicity.

**Fig. 1:**
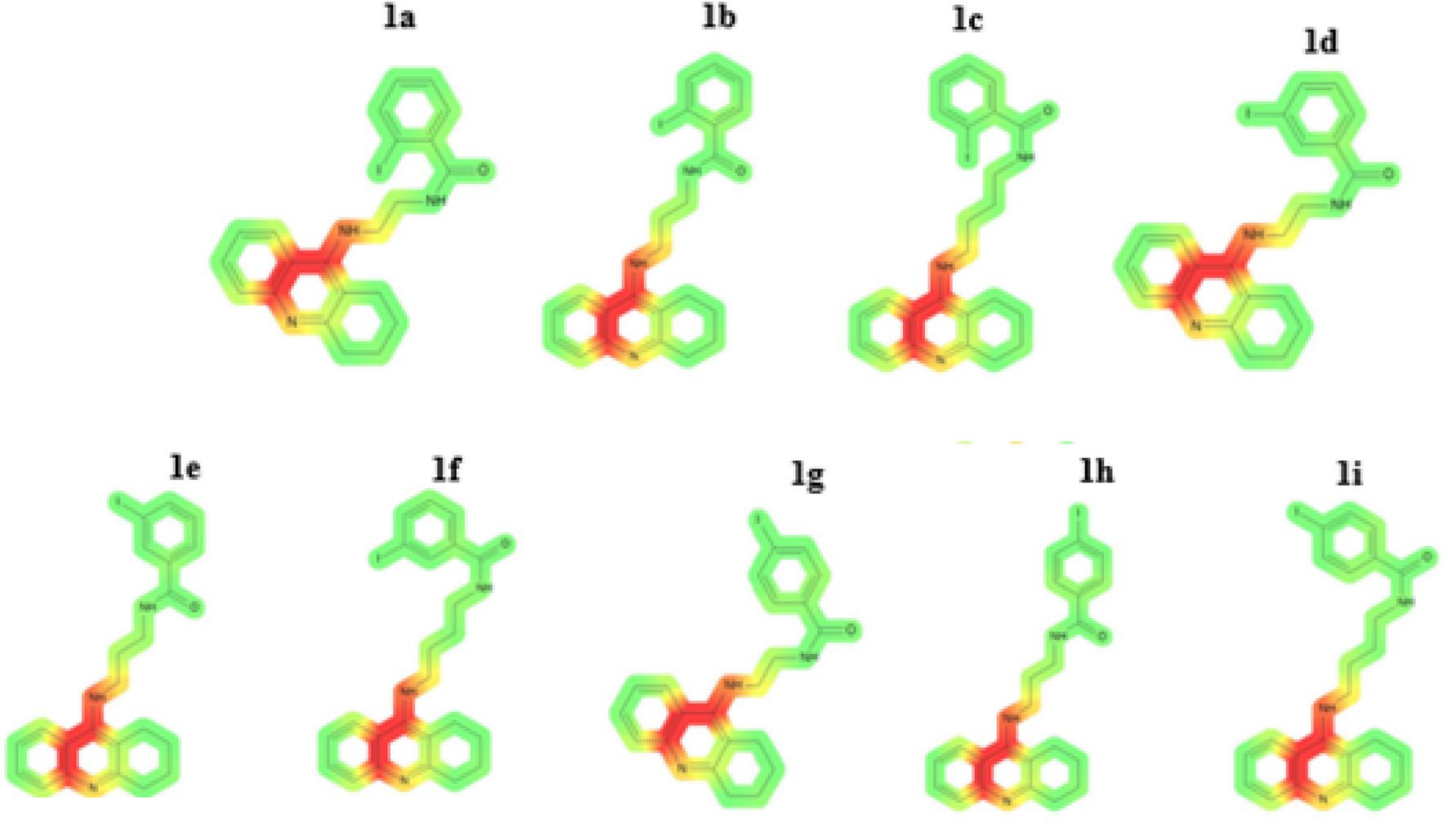
Visualization of genotoxic properties of compounds **1a**–**1i**. Genotoxic fragments are coloured in red. (Color figure online)

In order to evaluate potential mutagenicity of our compounds, the Ames test was performed. In general, Ames test is a validated in vitro test, in which histidine-dependent strain *Salmonella typhimurium* is exposed to a potential mutagen. Frame-shift mutation or base-pair substitutions are commonly observed. The in vitro Ames test is referred to the in silico calculations. Among novel compounds, test showed that compounds **1b**, **1e**, **1h**, with three carbons linker, had lowest mutagenic potential (Table 1). The length of the carbon chain did not influence on mutagenic potential of novel compounds [51]. Visualization of genotoxicity is presented in Fig. 1.

hERG (human ether-a-go-go-related gene) codes a protein which is a subunit of potassium ion channel. Some drugs inhibit hERG and can cause arrhythmias associated with lengthened QT interval (*torsades de pointes* and the risk for the sadden death). These drugs should be better examined for preclinical and clinical cardiovascular safety assessments [52]. The model calculates the probability of the new compound being a hERG inhibitor. In that case, IC_50_ is lower than the cut-off value of 10 µM. The results are presented as values: 1—hERG inhibitor, 0—non-inhibitor [53]. All of our novel compounds had values in the range of 0.51–0.56. Again, compounds **1b**, **1e**, **1h** with three carbons linker were the safest ones. The length of the carbon chain did not influence on interaction with hERG.

*P*-Glycoprotein (*P*-gp) is known as a multidrug resistance protein 1 (MDR1), belongs to the ATP Binding Cassette Superfamily [54]. *P*-gp is located in the intestinal epithelium, hepatocytes, renal proximal tubular cells, adrenal glands and capillary endothelial cells [34, 55]. Most of *P*-gp substrates are hydrophobic, therefore *P*-gp is called ‘hydrophobic vacuum cleaner’ and transports substance across extra- and intracellular membranes, extrudes compounds (structurally unrelated hydrophobic substances, pollutants and drugs) from inside to outside of cells. *P*-gp transport of novel compounds limiting their bioavailability. In the ADMET calculation, drugs can be classified as *P*-gp substrate or inhibitor. There is a correlation between *P*-gp overexpression and MDR resistance. *P*-gp overexpression is found in various cancer cells which develop increased efflux activity. Unfortunately, many clinically anticancer agents (such as etoposide) are substrates of *P*-gp [56]. *P*-gp substrates have reduced bioavailability after oral administration and decreased ability to cross blood–brain barrier. It can cause impairment in effective treatment outcomes [54, 57–59]. Compounds **1a**, **1d** and **1g** had the lowest ability to be *P*-gp inhibitor or substrate. The position of iodine in *ortho* position influenced the interaction with *P*-gp, whereas the length of the carbon chain did not.

The volume of distribution, the distribution of compounds between plasma and body tissue (*V*_d_), the cumulative percentage of a compound bound to human plasma proteins (%PPB) and the human serum albumin affinity constant (log *K*_a_^HSA^) were calculated [34]. The *V*_d_ is general calculated from measurements of the total concentration of drug in the blood compartment after a single intravascular injection. Physical properties of the drug influence on *V*_d_ values. Acid drugs bind easier to plasma proteins and have smaller *V*_ds_ than basic drugs, which bind more extravascular proteins and have larger *V*_ds_. Larger *V*_d_ indicates extravascular binding or storage in fat or other tissues. *V*_d_ = 5 L—distribution is limited to blood; *V*_d_ = 10–20 L—drug penetration into extracellular fluids; Vd = 25–35 L—drug penetration into intracellular fluids; *V*_d_ = 40 L—placement in all liquids of the body; *V*_d_ > 100 L—the drug is strongly concentrated in the tissues. The iodo position in *para* position and longer carbon chain increased *V*_d_ values. All inhibitors, except **1a**, **1b** and **1c,** had *V*_d_ values above 10 L, which indicated penetration of the compounds into extracellular fluids. The most important aspect of the drug distribution from plasma to target tissue is %PPB. Compounds bind to the blood proteins such as albumin (acidic drugs—warfarin, phenytoin), globulin α and β (vitamins A, D, E, K, B_12_, hormones), acid a1-glycoprotein, lipoproteins—(drugs with basic character—propranolol, lidocaine). The clinical significance is observed when degree of drug binding with protein is greater than 80%. Drug binding to protein is inactive pharmacologically; cannot pass through biological membranes; is not metabolized and cannot be excreted. Drugs with high degree of binding are for example coumarin derivatives, phenylbutazone, salicylates, sulphonamides or penicillin. Reducing the degree of drug binding results in increasing the potency of the drug and shortening of drug action time. All novel compounds had very high %PPB—between 96 and 97%. These values increased within iodine position in the benzene ring. The highest %PPB was observed for iodine in *para* position. The human serum albumin affinity constant was also calculated for all compounds. The highest affinity had compounds with three or four carbons linker [34].

### In vitro cytotoxicity of new compounds against cancer and non-cancer cell lines

**Fig. 2:**
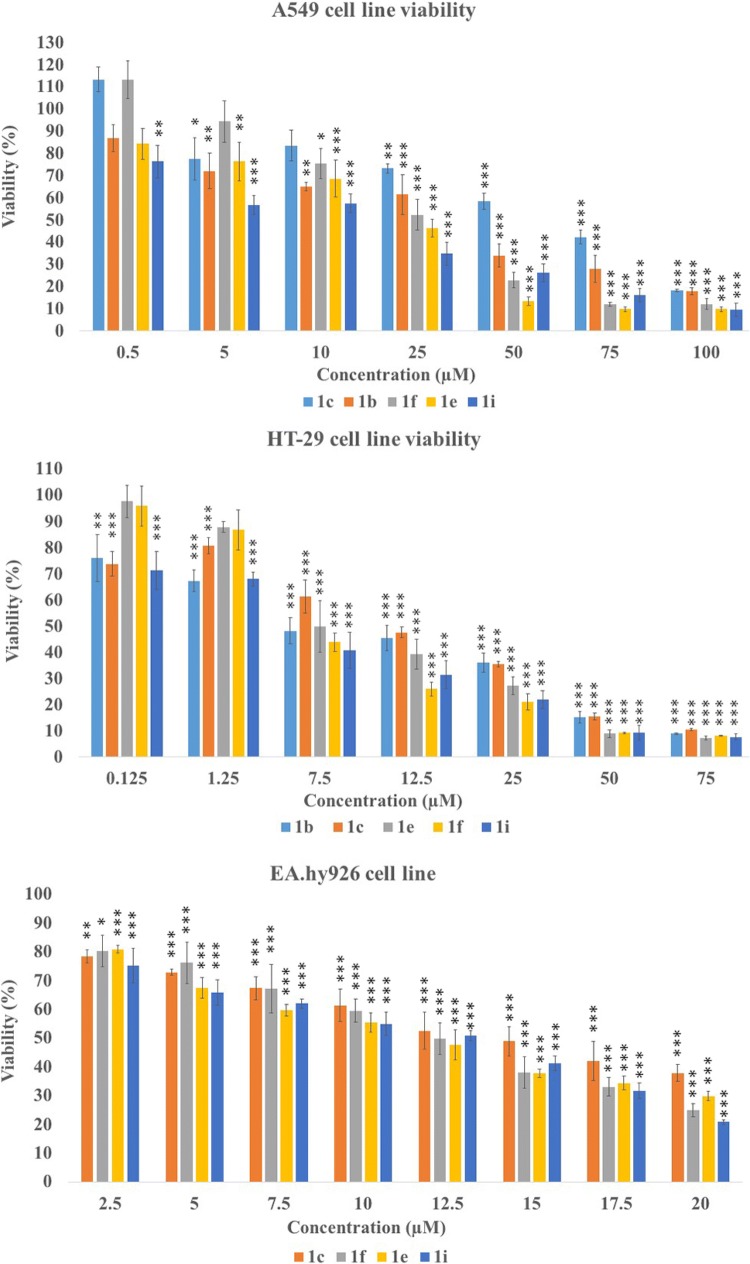
Effect of selected compounds on the viability of A549 cells, HT-29 cells and EA.hy926 cells. Statistical significance was assessed using one-way ANOVA with a post hoc analysis was performed. ***p < 0.001, **p < 0.01, *p < 0.05 was considered as significantly different in comparison to non-treated control

The tetrahydroacridine derivatives with iodobenzoic acid moiety **1a**–**1i** were tested in vitro by MTT assay against two human cancer cell lines—lung adenocarcinoma A549 and colorectal adenocarcinoma HT-29; and one human non-cancer cell line—umbilical vein EA.hy926. Concentration–response analysis was used to determine IC_50_ values. The results are shown in Table 2 and Fig. 2. Widely used clinical chemotherapeutic agents— etoposide and 5-FU were used as positive controls. Etoposide is used to treat lung cancer, whereas 5-FU is used in the treatment of colorectal cancer. All tested tetrahydroacridine derivatives showed cytotoxicity activity with the IC_50_ values in the range from 59.12 µM to 5.90 µM against cancer cell lines. Controls compounds possessed lower cytotoxicity activity than tested derivatives—etoposide IC_50_ 451.47 µM, 5-fluorouracil IC_50_ 1626.85 µM. For A549 cell line, IC_50_ values were higher than for HT-29 cells, which indicated that colorectal adenocarcinoma cells were more sensitive to tetrahydroacridine derivatives than lung adenocarcinoma cells. **1i** (IC_50_ 14.87 µM against A549 and 5.90 µM against HT-29) was the most cytotoxic compound versus lung and colorectal adenocarcinoma cells. Compounds were divided into three groups dependently on the iodine position. **1a**–**c**—iodine in *ortho* position, **1d**–**f**—iodine in *meta* position and **1g**–**i**—iodine in *para* position. Against lung and colorectal cancer cells, the structure–activity relationship showed that longer carbon linker much increased cytotoxic activity. All group of *para* position compounds had the strongest cytotoxic effect. The position of iodine in the substituent in the *para* position and 4 carbon linkers most strongly enhanced the cytotoxic effect, what was observed for IC_50_ values for compound **1i**. Compounds with the highest cytotoxicity and longer linker (3 or 4 carbons) were chosen for next biological tests. Since, the injury of endothelium is one of the side effects during chemotherapy, the most active compounds **1b**, **1e**, **1i** towards A549 cell line and the most active compounds **1c**, **1f**, **1i** against HT-29 cells were chosen to determine their cytotoxicity against normal endothelial cell line—EA.hy926. **1c**, **1f**, **1i** presented slightly lower cytotoxicity effects towards EA.hy926 cells compared to colorectal cancer cells (**1c** IC_50_ 13.58 µM, 14.32 µM—no statistically significant; **1f** IC_50_ 8.52 µM, 12.36 µM statistically significant; **1i** IC_50_ 5.90 µM, 11.30 µM statistically significant—against HT-29 and EA.hy926, respectively). Whereas the most toxic compounds **1b**, **1e**, **1i** presented higher toxicity for EA.hy926 cells compared to A549 lung cancer cells (**1b** IC_50_ 32.52 µM, 22.42 µM; **1f** IC_50_ 23.49 µM, 11.72 µM; **1i** 14.87 µM, 11.30 µM—against A549 and EA.hy926 cells, respectively). These results suggested that compounds were more toxic towards HT-29 colorectal cancer cells than normal cells—EA.hy926. On the contrary, etoposide showed higher cytotoxic activity towards EA.hy926 cells (IC_50_ 155.19 µM) than A549 cells (IC_50_ 451.47 µM), according to the data reported by [9]. Finally, 5-fluorouracil presented similar cytotoxicity against HT-29 and EA.hy926 cells.

**Table 2:** In vitro cytotoxic activity of compounds **1a**–**1i** and reference compounds on two cancer cell lines (A549 and HT-29) and one non-cancer cell line (EA.hy926)

Compound	Moiety	Number of carbons	IC_50_ µM ± SD against A549	IC_50_ µM ± SD against HT-29	IC_50_ µM ± SD against EA.hy926
**1a**	2-Iodobenzoic acid	2	35.96 ± 1.74	17.32 ± 1.22	–
**1b**	2-Iodobenzoic acid	3	32.52 ± 2.25**	16.08 ± 1.55*	22.42 ± 2.30
**1c**	2-Iodobenzoic acid	4	59.12 ± 5.86**	13.58 ± 1.39	14.32 ± 1.65
**1d**	3-Iodobenzoic acid	2	41.16 ± 1.27	7.62 ± 0.19	–
**1e**	3-Iodobenzoic acid	3	23.49 ± 2.60**	11.03 ± 0.59	11.72 ± 0.62
**1f**	3-Iodobenzoic acid	4	26.43 ± 2.45**	8.52 ± 0.10**	12.36 ± 1.31
**1g**	4-Iodobenzoic acid	2	20.79 ± 1.58	8.16 ± 0.44	–
**1h**	4-Iodobenzoic acid	3	21.36 ± 1.54	9.40 ± 1.65	–
**1i**	4-Iodobenzoic acid	4	14.87 ± 1.06*	5.90 ± 0.42**	11.30 ± 0.89
Etoposide			451.47 ± 18.27**		155.19 ± 9.81
5-Fluorouracil				1626.85 ± 49.26	> 1800 µM

All values are presented as the mean ± SD; IC_50_, 50% inhibition of the cell viability. Statistical significance was assessed using one-way ANOVA analysis was performed

*p < 0.05; **p < 0.01 was considered as significantly different between cancer and non-cancer cell line

### Colony forming assay

**Fig. 3:**
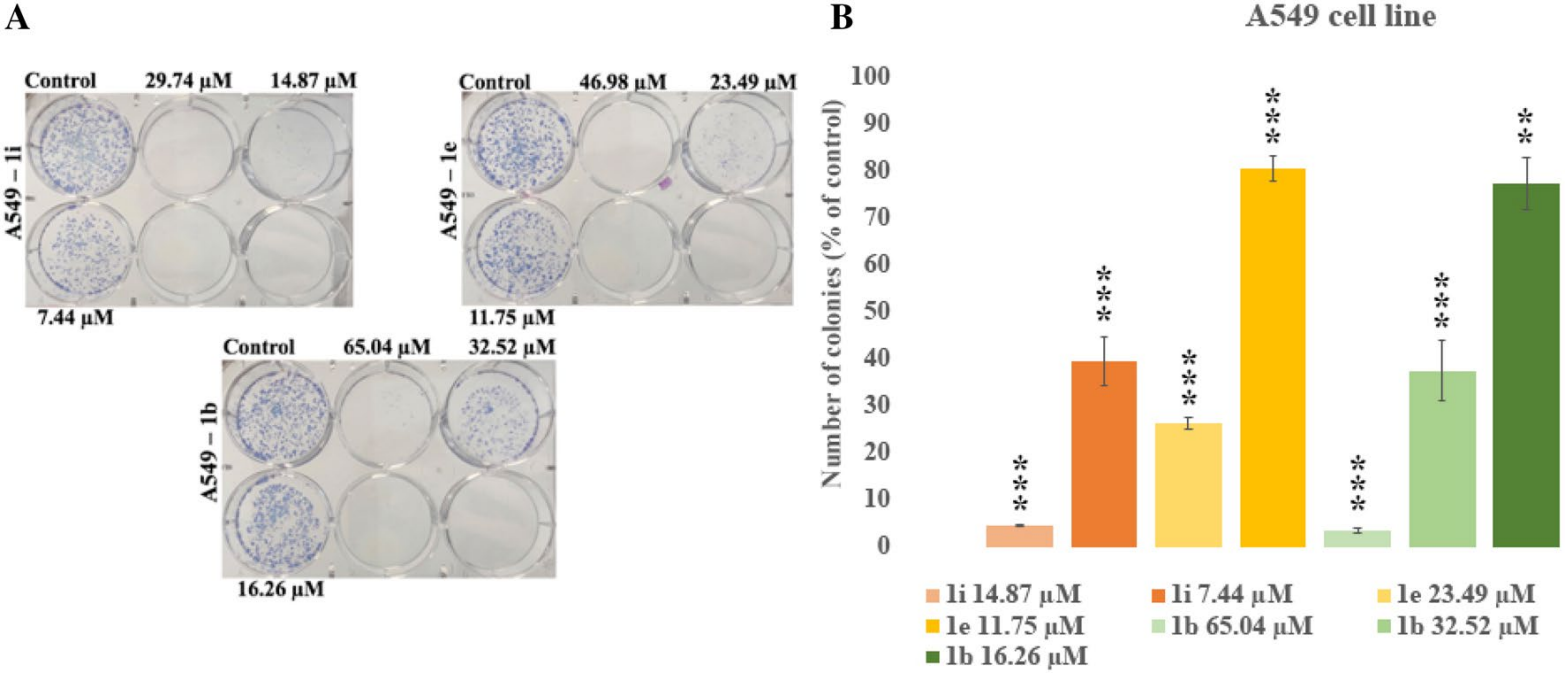
**a** Representative colony forming assay of A549 cell line. **b** Number of colonies expressed as percent of control number. Statistical significance was assessed using one-way ANOVA with a post hoc analysis was performed. ***p < 0.001, **p < 0.01, was considered as significantly different in comparison to non-treated control

**Fig. 4:**
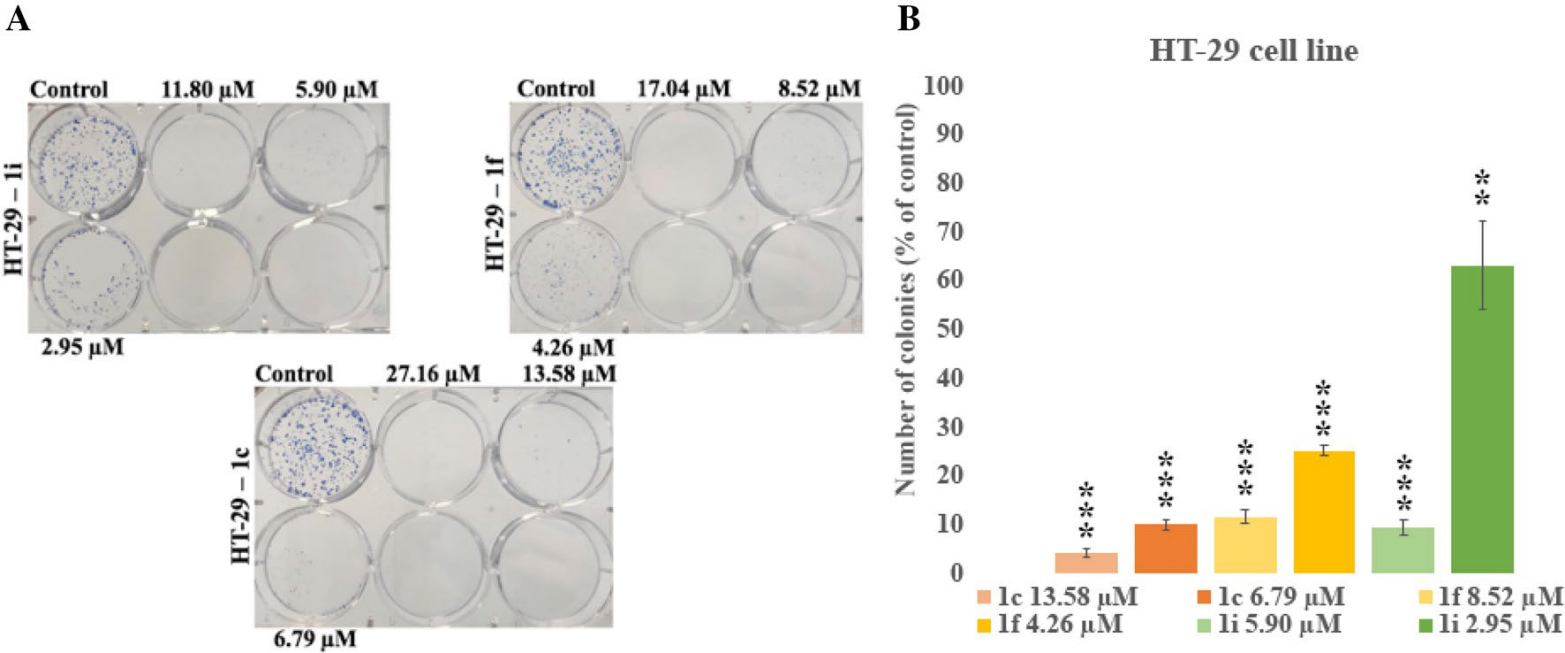
**a** Representative colony forming assay of HT-29 cell line. **b** Number of colonies expressed as percent of control number. Statistical significance was assessed using one-way ANOVA with a post hoc analysis was performed. ***p < 0.001, **p < 0.01, was considered as significantly different in comparison to non-treated control

The anticancer properties of novel tetrahydroacridine derivatives were tested in the clonogenic assay. Compounds **1b**, **1e**, **1i** at their 2xIC_50_, IC_50_ and _1/2_IC_50_ concentrations were incubated with A549, whereas compounds **1c**, **1f**, **1i** were tested in HT-29 cell line. All compounds decreased A549 colony growth in a concentration-dependent manner (Fig. 3). For A549 cells, compound **1i** showed the lowest number of colonies (highest cytotoxic effect) at its 2xIC_50_ (0%), IC_50_ (4.70%) and _1/2_IC_50_ (39.47%) concentrations in comparison to the same concentrations of compounds **1b** and **1e**. The results were comparable to the results from MTT assay, where compound **1i** was the most cytotoxic against A549 cells. In the test with HT-29 cells, compound **1c** was the most cytotoxic compound at its 2xIC_50_ (0%), IC_50_ (4.38%) and _1/2_IC_50_ (10.06%) concentrations in comparison with **1f** and **1i** (Fig. 4). At the concentration of 2xIC_50_, no cells of A549 and HT-29 made colonies. At this concentration the strongest apoptotic effect was observed. The only exception was compound **1b**, for which at 2xIC_50_ concentration, cell made small number of colonies (3.55% in comparison to the control). Higher cytotoxic effect was observed for HT-29 cells than for A549 cells, similar like in the MTT assay.

### Cell cycle arrest analysis

**Fig. 5:**
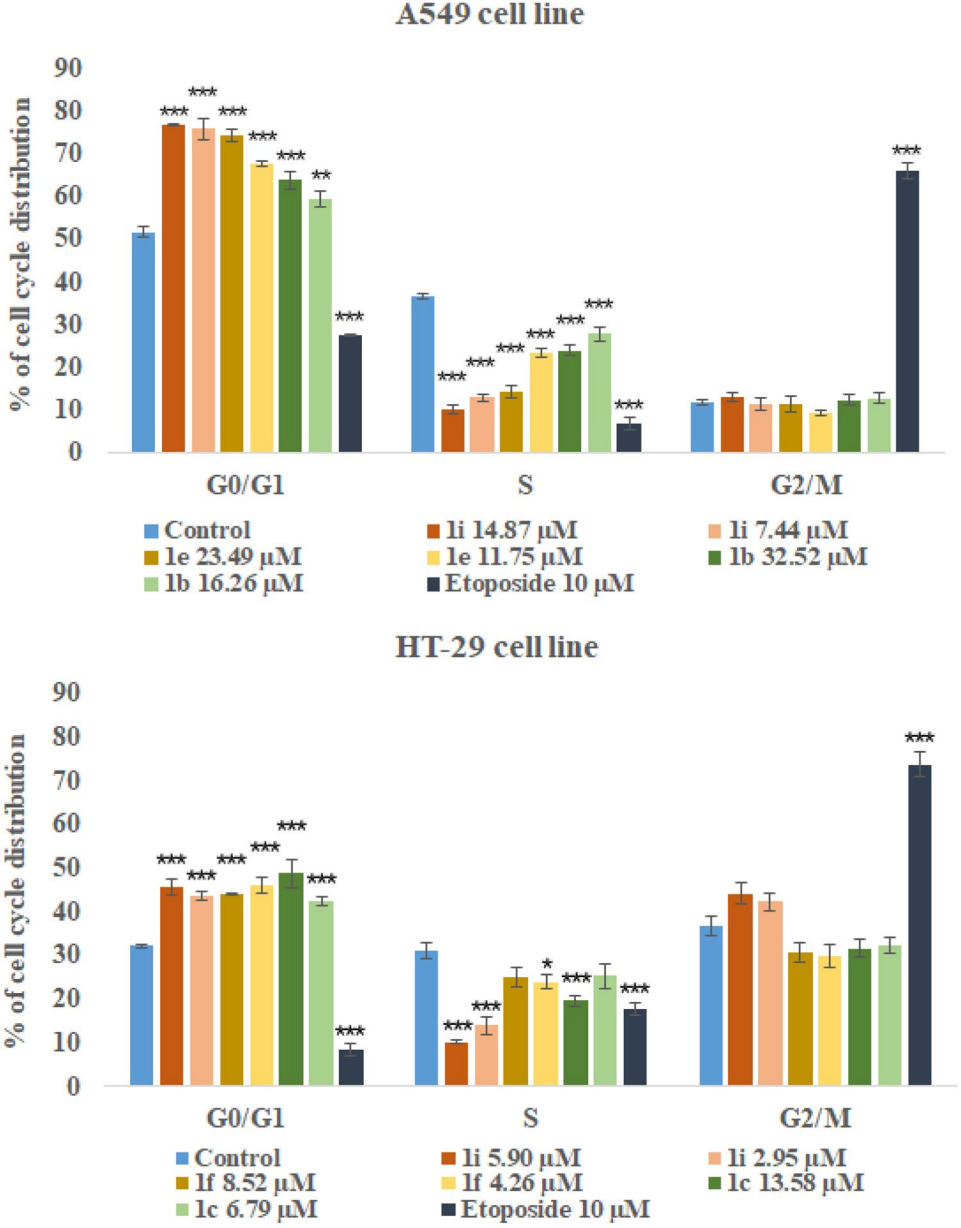
Percentage of cell cycle distribution after 24 h treatment of A549 cells with compounds **1b**, **1e**, **1i** at their IC_50_ and _1/2_IC_50_ concentrations. Percentage of cell cycle distribution after 24 h treatment of HT-29 cells with compounds **1c**, **1e**, **1i** at their IC_50_ and _1/2_IC_50_ concentrations. Etoposide was used as a reference compound. Experiment was repeated three times. Data were presented as the mean ± SD. One-way ANOVA analysis with post hoc analysis was performed. ***p < 0.001, **p < 0.01, *p < 0.05 was considered as significantly different in comparison to non-treated control

**Fig. 6:**
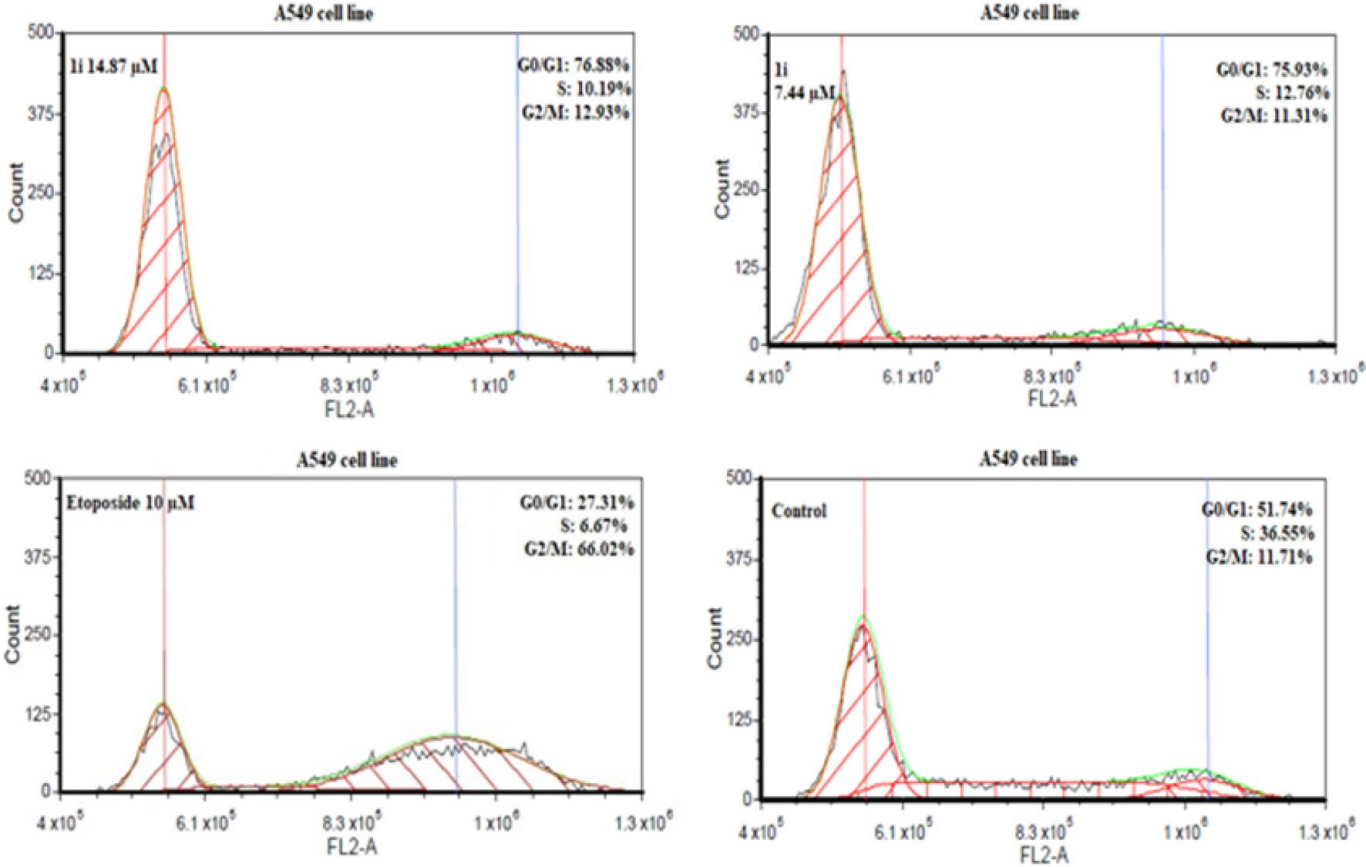
A549 cells were treated with the most active compound **1i** at its IC_50_ and _1/2_IC_50_ concentrations for 24 h. Etoposide (10 µM) was used as a reference compound. Analysis of DNA content was performed by using flow cytometry after PI staining

**Fig. 7:**
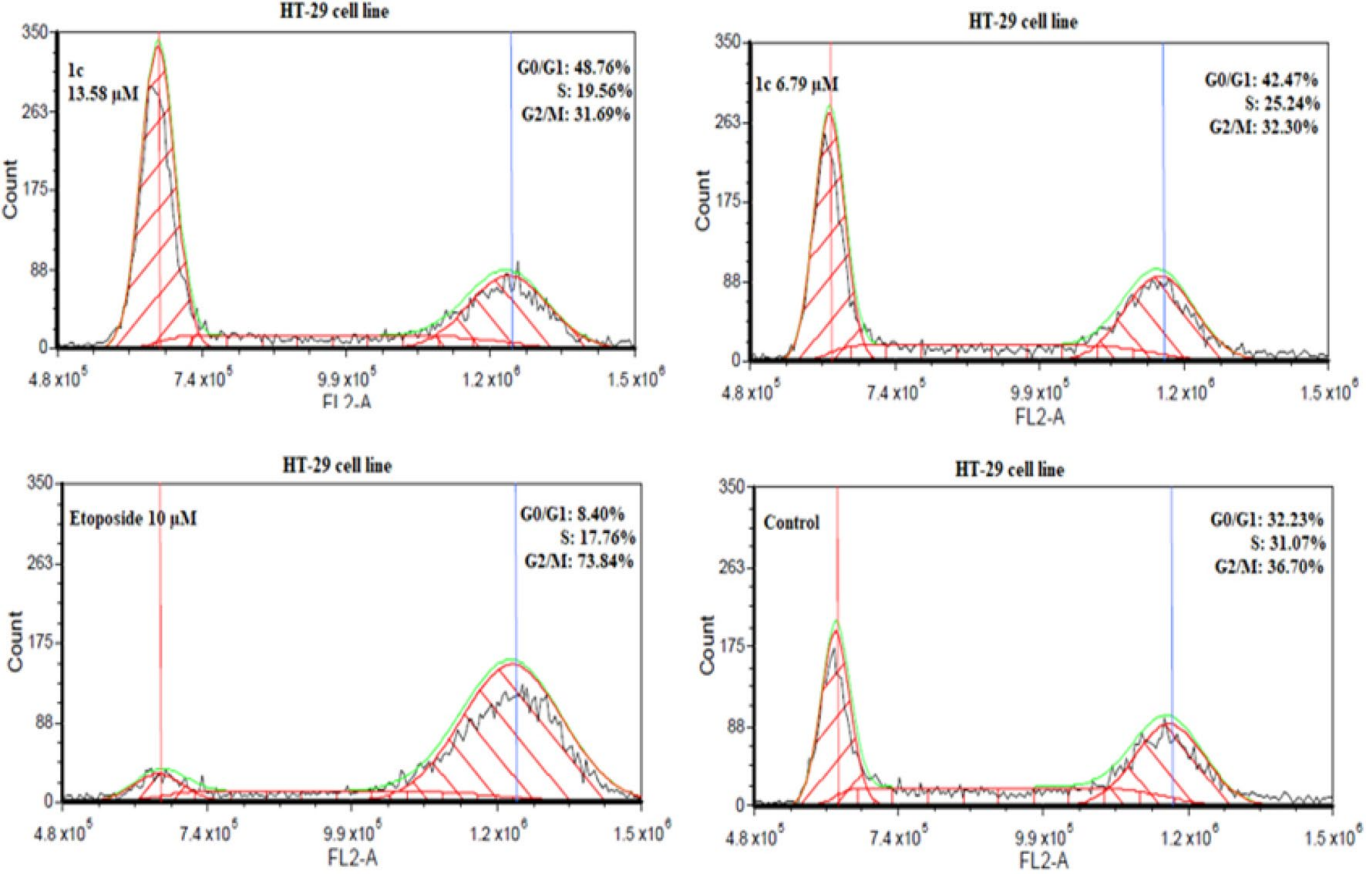
HT-29 cells were treated with the most active compound **1c** at its IC_50_ and _1/2_IC_50_ concentrations for 24 h. Etoposide (10 µM) was used as a reference compound. Analysis of DNA content was performed by using flow cytometry after PI staining

The ability of tetrahydroacridine derivatives to impair cell proliferation could depend on the block of cell cycle progression and/or on the induction of apoptosis. To clarify the mechanisms of action of these compounds, a flow cytometric analysis of cell cycle distribution of A549 and HT-29 cells after incubation with our derivatives was performed. Date were analysed by FCS Express 4 Flow Cytometry Software. Compounds **1b**, **1e**, **1i** at their IC_50_ and _1/2_IC_50_ concentrations were chosen to evaluate cell cycle arrest in A549 cell line, whereas compounds **1c**, **1f**, **1i** were tested in HT-29 cell line. Etoposide was used as a reference compound at the concentration of 10 µM. Compounds **1b**, **1e**, **1i** induced a significant accumulation of cells G0/G1 phase in comparison to control, causing a decrease in S phase cells in concentration-dependent manner (Fig. 5) Phase G0/G1 accumulation was induced by all compounds in comparison to control. The highest values were observed for **1i** (14.87 µM 76.88%, 7.44 µM 75.93%, control cells 51.74%) (Fig. 6). Results were in agreement with those obtained from MTT test, where, **1i** was the most cytotoxic compound for A549 cells. Results obtained in HT-29 cells, confirmed that novel tetrahydroacridine derivatives induced an accumulation of G0/G1 phase cells. However, this effect was smaller than that observed in A549 cells. Among compounds, **1c**, **1f**, **1i**—compound **1c** was the most effective (13.58 µM 45.59%, 6.79 µM 43.75%, non-treated cells 32.23%) (Fig. 7). Etoposide (10 µM) induced a G2/M accumulation in both cell lines (66.02% accumulation of A549 cells and 73.84%. in HT-29 cells).

### Apoptosis assay by annexin-V/PI double staining

**Fig. 8:**
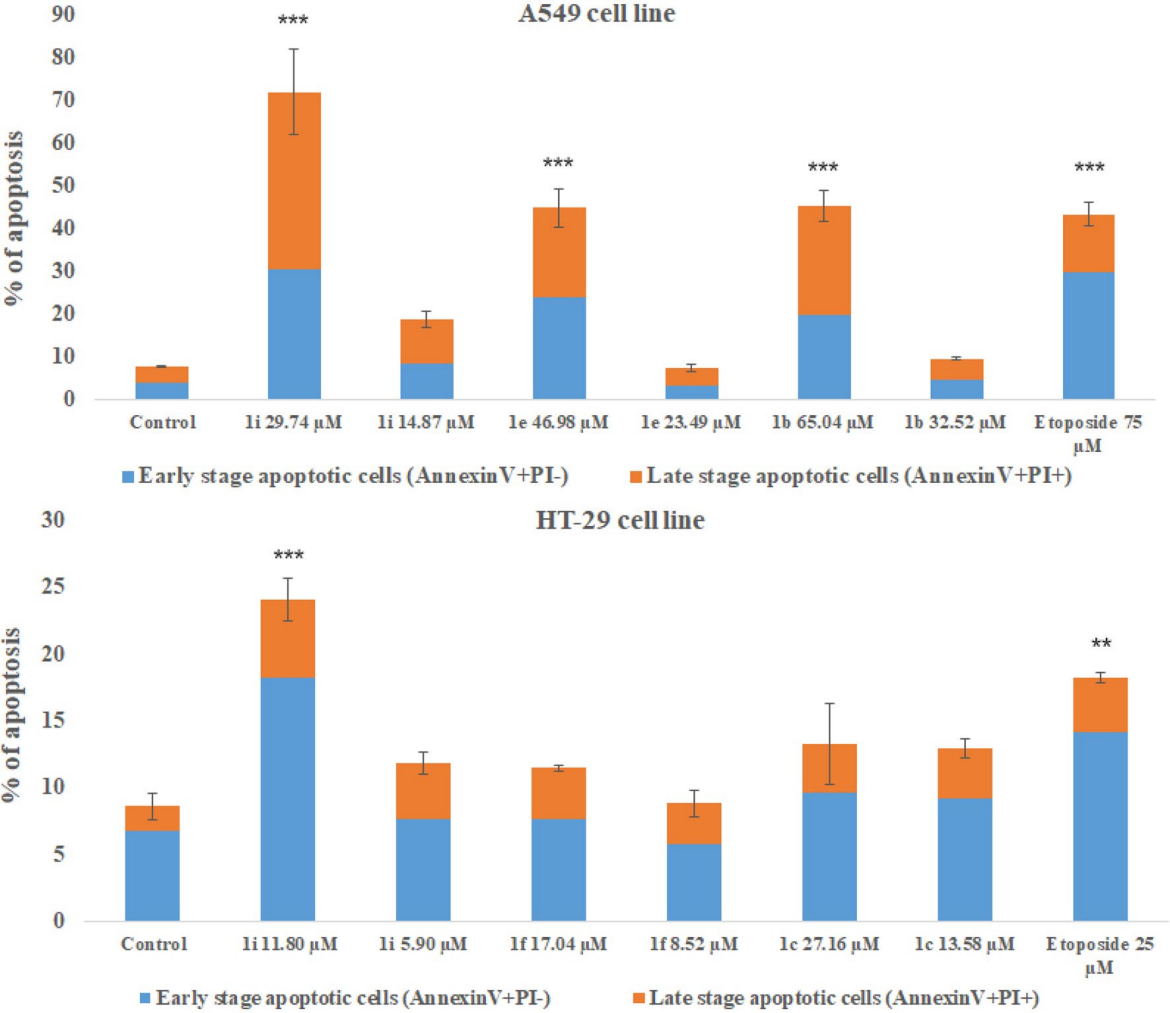
Cytofluorimetric analysis of apoptosis in A549 and HT-29 cells after treatment with chosen compounds **1b**, **1e**, **1i** (A549 cells) and **1c**, **1f**, **1i** (HT-29 cells) after 24 h of incubation. Data were presented as the mean ± SD and expressed as percentage of total apoptosis. Experiment was repeated three times. One-way ANOVA analysis with post hoc analysis was performed. ***p < 0.001, **p < 0.01 were considered as significant in comparison to non-treated control

**Fig. 9:**
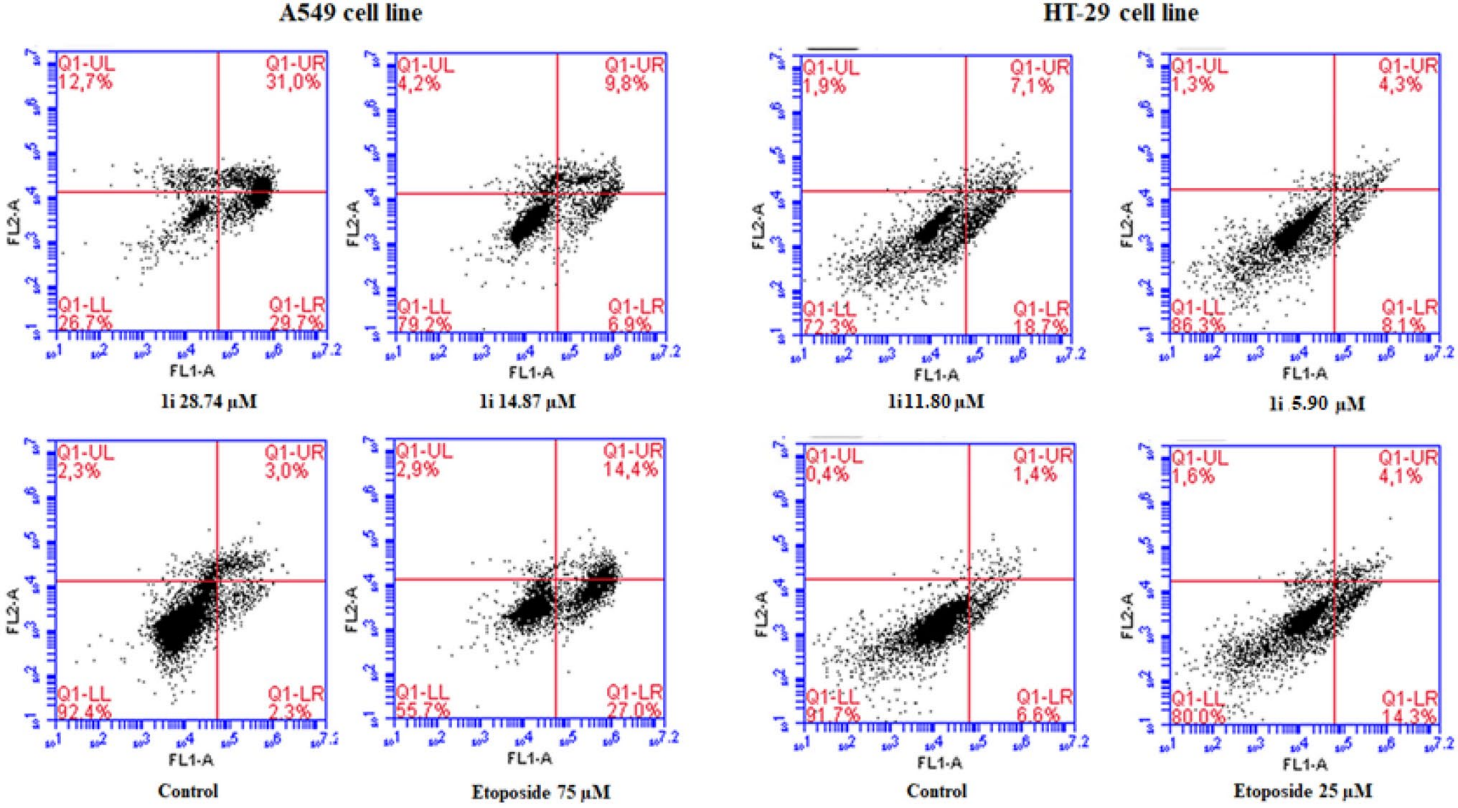
A549 cells and HT-29 were treated with the most active compound **1i** at its 2xIC_50_ and IC_50_ concentrations for 24 h. Etoposide was used as a reference compound

Apoptosis is a process of programmed cell death, which is necessary to maintain proper homeostasis. Physiological apoptosis is responsible for controlling cell numbers, morphology of organs and tissue or removal of injured and mutated cells. Pathological apoptosis leads to cancers (and further to resistance of chemotherapy), neurodegenerative and hyperproliferative disorders [60–62]. Important goal of anticancer drugs is to induce apoptosis in tumour cells. Annexin-V/PI double-staining assay was performed to investigate whether our novel tetrahydroacridine derivatives were able to induce apoptosis [39, 63]. Results were analysed by using flow cytometry. Compounds **1b**, **1e**, **1i** at their 2xIC_50_ and IC_50_ concentrations were chosen to inducement apoptosis in A549 cell line, whereas compounds **1c**, **1f**, **1i** were tested in HT-29 cell line. All compounds induced apoptotic effect in a concentration-dependent manner. In control of A549 cells, the mean percentage of total apoptotic cells was 7.80%. Compound **1i** caused the strongest apoptotic effect among compounds tested in A549 cells. After treatment with **1i**, at the concentration of 29.74 µM, apoptotic cells were 71.98%. For the concentration of 14.87 µM, apoptotic cells were 18.79%. Higher apoptotic effect was observed for double concentration of IC_50_. Compounds **1e** and **1b** also induced a high level of apoptosis at 2xIC_50_ (by 44.96% and 45.31%, respectively). Etoposide at the concentration of 75 µM—caused total apoptosis at the level of 43.46% (Fig. 8). In Fig. 9, plots were divided into four quadrants. Each quadrant represents difference type of cells: living cells (annexin-V−/PI−, lower left quadrant, LL), early apoptotic cells (annexin-V+/PI−, lower right quadrant, LR), late apoptotic cells (annexin-V+/PI+, upper right quadrant, UR). Among all compounds tested, in HT-29 cell line, total apoptotic cells for control were 8.62%. For HT-29 cells, compound **1i** induced the strongest apoptotic effect. At the concentration of 11.80 µM, apoptotic cells were 24.10%. At the IC_50_ concentration—5.90 µM, apoptotic cells were 11.80%. However, slightly higher apoptotic effect at IC_50_ was observed for **1c**—12.96%. Compound **1i** caused higher apoptosis in A549 cells than in HT-29 cells. Etoposide at the concentration of 25 µM, caused a total apoptotic effect by 18.18% (Fig. 8).

### DNA unwinding assay

Intercalation of molecules into DNA has been studied for many years in order to study novel drugs and gene expression. Intercalator has polycyclic, aromatic structure, which can insert between bases of the double-helical DNA. Acridine and its derivatives are well-known intercalators, which binds reversibly to DNA, but usually not covalently. Intercalation of acridines is possible due to the cationic ionization and molecular planarity. Compounds, which intercalate into DNA, lead to local unwinding of the DNA and decrease in the twist of DNA. In the presence of topoisomerase, DNA is nicked and rejoined, which results in its relaxation. Removal of the enzyme and tested compound leads to conversion relaxed DNA to supercoiled DNA. This formation of supercoiled DNA is an indication that compound is an intercalator [43].

**Fig. 10:**
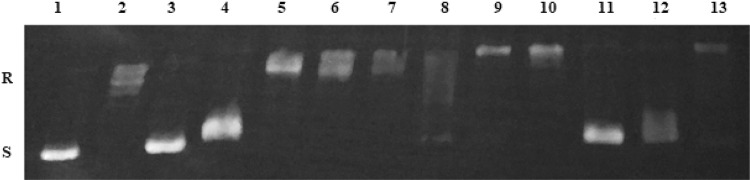
Lane 1—supercoiled pUC19 DNA; lane 2—pUC19 DNA + TopoI—resulted in relaxation of plasmid, lane 3—pUC19 DNA + TopoI + amsacrine (100 µM); lane 4—pUC19 DNA + TopoI + EB (100 µM); lane 5, 6, 7—pUC19 DNA + TopoI + **1i** 100 µM, 14.87 µM, 5.90 µM, respectively; lane 8, 9, 10—pUC19 DNA + TopoI + **1f** 100 µM, 26.43 µM, 8.52 µM, respectively; lane 11, 12, 13—pUC19 DNA + TopoI + **1c** 100 µM, 59.12 µM, 13.58 µM, respectively. Lanes 1–4 are control lanes. Lanes 5–12 are results lanes of tested compounds. R—relaxed pUC19 DNA. S—supercoiled pUC19 DNA

**Fig. 11:**
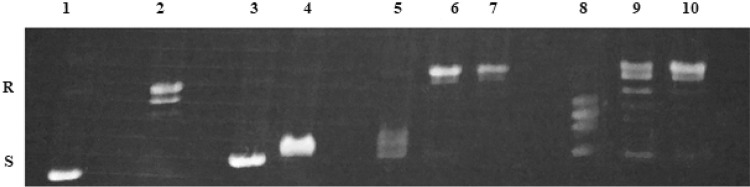
Lane 1—supercoiled pUC19 DNA; lane 2—pUC19 DNA + TopoI resulted in relaxation of the plasmid, lane 3—pUC19 DNA + TopoI + amsacrine (100 µM); lane 4—pUC19 DNA + TopoI + EB (100 µM); lane 5, 6, 7—pUC19 DNA + TopoI + **1e** 100 µM, 23.49 µM, 11.03 µM, respectively; lane 8, 9, 10—pUC19 DNA + TopoI + **1b** 100 µM, 32.52 µM, 16.08 µM, respectively. Lanes 1–4 are control lanes. Lanes 5–10 are results lanes of tested compounds. R—relaxed pUC19 DNA. S—supercoiled pUC19 DNA

The ability of compound to bind into DNA, which leads to unwinding of the DNA, was determined in DNA unwinding assay catalyzed by human Topoisomerase I. It was checked, whether additional moiety influence on intercalation process. Compounds **1b**, **1c**, **1e**, **1f**, **1i** were tested in every three concentrations: 100 µM, IC_50_ of A549, IC_50_ of HT-29. In the agarose gel, depending on shape, supercoiled plasmid DNA (S) migrated further, whereas relaxed DNA (R) moved shorter distance. EB was not added to the agarose gel. In Fig. 10 and 11, lane 2 shows that supercoiled pUC19 DNA was relaxed by the TopoI during incubation without any compound. If compound is not an intercalator relaxed plasmid is also a product of reaction. Amsacrine, an inhibitor of human topoisomerase II, and EB, only an intercalator, were used as reference compounds. Results showed, that amsacrine (100 µM) was stronger intercalator than EB (100 µM). **1b**, **1c**, **1e**, **1f** at the concentration of 100 µM, had bands at the same level as EB. Only **1i**, which was the strongest cytotoxic compound in the MTT assay, did not have any bands at supercoiled level. Among all compounds, the compound **1c** had the best intercalative potential at concentrations of 100 µM and 59.12 µM. Results showed, that four from five tetrahydroacridine derivatives could intercalate into DNA and induced supercoiled form of DNA.

### Western blotting studies of the expression of PARP-1 and γ-H2AX

The induction of apoptosis by tetrahydroacridine derivatives was also investigated through the analysis of poly(ADP Ribose) polymerase-1 (PARP-1) cleavage, which is considered as a hallmark of apoptosis. PARP-1 is a 116 kDa protein, which has a wide range of physiological and pathological functions. PARP-1 detects and repairs damage DNA by binding to strand breaks, initiating base excision repair and nucleotide excision repair [64]. One of the crucial signalling pathways involved in apoptosis is the activation of caspases. During apoptotic process, caspases cleave several crucial proteins, which regulate cellular function and survival. PARP-1 is one of the substrates of caspases. PARP-1 is a target for cleavage by caspase-3 (which also cleaves PARP-1 in neurological diseases) and caspase-7. Caspases cleave PARP-1 between Asp214 and Gly215 residues in order to produce 89 kDa and 24 kDa fragments [65, 66], 89 kDa fragment contains auto-modification domain (AMD), and its catalytic domain reduces DNA binding ability (in non-cleaved PARP AMD induces the recruitment of DNA repairing enzymes) and is released from nucleus into cytosol. 24 kDa fragment with two zinc-finger motifs, stays inside nucleus where inhibits active PARP-1 and irreversibly binds to nicked DNA. Binding to strand breaks blocks DNA repair enzymes (active PARP-1 is inhibited), weakens DNA repair and preserve ATP pools in cell. When cells has tremendous DNA damage, PARP-1 is over-activated and uses a large number of NAD+. This activity leads to necrotic cell death [60, 67–69].

**Fig. 12:**
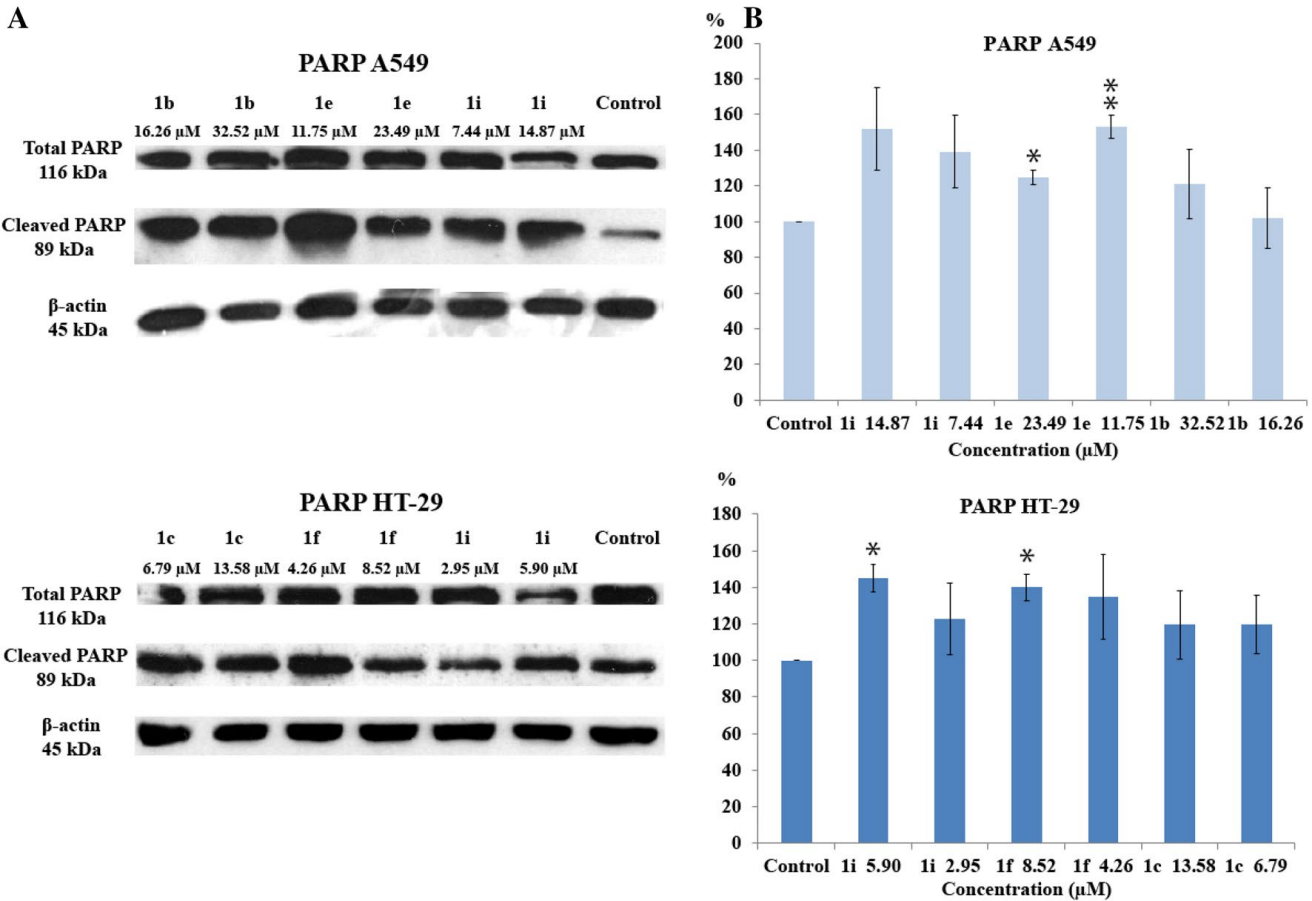
**a** Western blot analysis of total (116 kDa) and cleaved (89 kDa) PARP-1 proteins levels in A549 and HT-29 cells after 24 h. Cells were treated with tetrahydroacridine derivatives at IC_50_ and _1/2_IC_50_ concentrations. **b** Relative densitometric values of cleaved PARP-1 proteins levels. Protein quantification was performed by densitometric scanning. Experiment was repeated at least two times. Data were normalized using β-actin, indicated as mean ± SD and expressed as a percentage of control. One-way ANOVA analysis with post hoc analysis was performed. **p < 0.01, *p < 0.05 were considered as significant in comparison to non-treated control

Tetrahydroacridine derivatives were tested in inducing PARP-1 cleavage. Compounds **1b**, **1e**, **1i** were tested in A549 cells, whereas **1c**, **1f**, **1i** were used in HT-29 cells—all compounds were investigated at their IC_50_ and _1/2_IC_50_ concentrations. Cleaved PARP-1 protein (89 kDa) was observed in all samples after treatment of A549 cells. The highest effect at its IC_50_ of each compound was observed after treatment with **1i** at 14.87 µM. Compound **1b** and **1e** had comparable apoptotic effect at their IC_50_ concentrations (23.49 µM, 32.52 µM, respectively). However, the highest PARP-1 cleavage was observed for _1/2_IC_50_ (11.75 µM) of **1e**. In HT-29 cells, induction of PARP-1 cleavage was lower than for A549 cells Compound **1c** and **1f** induced lower apoptosis at IC_50_ concentrations (13.58 µM, 8.52 µM, respectively) than **1i**. Similar like A549 cells, the highest PARP-1 cleavage was observed for **1i** at the IC_50_ concentration (5.90 µM) (Fig. 12). All tested tetrahydroacridine derivatives induced PARP-1 cleavage confirming the apoptosis induction.

Our results confirmed that novel tetrahydroacridine derivatives caused cytotoxicity, inhibited cell cycle progression in G0/G, induced apoptosis and intercalated to DNA. Next analysis was performed to determine molecular mechanism of cell death through western blot analysis of γ-H2AX expression. It is well known that double-stranded breaks (DSBs) of DNA followed by phosphorylation of histone H2AX (γ-H2AX). H2AX belongs to family of H2A proteins. Histone proteins H2A, H2B, H3, H4 form a core, around which DNA is wrapped to make a nucleosome complex. H2AX is an important element in the repairing process of damaged DNA. It connects to damage sites, which activates other DNA repairing processes. H2AX protein is unique because of its carboxy tail, which comprises serine at position 139. Phosphorylation (by kinase ataxia telangiectasia mutated (ATM)) on the 139th serine residue occurs during DNA damage (γ-H2AX) and after repairing, γ-H2AX is dephosphorylated. γ-H2AX is studied by many research’s teams in the context of cancer treatment. Numerous cytotoxic agents, such as camptothecin, cisplatin, doxorubicin, etoposide or bleomycin are known as agents which cause DNA damage and formation of γ-H2AX [70–72].

**Fig. 13:**
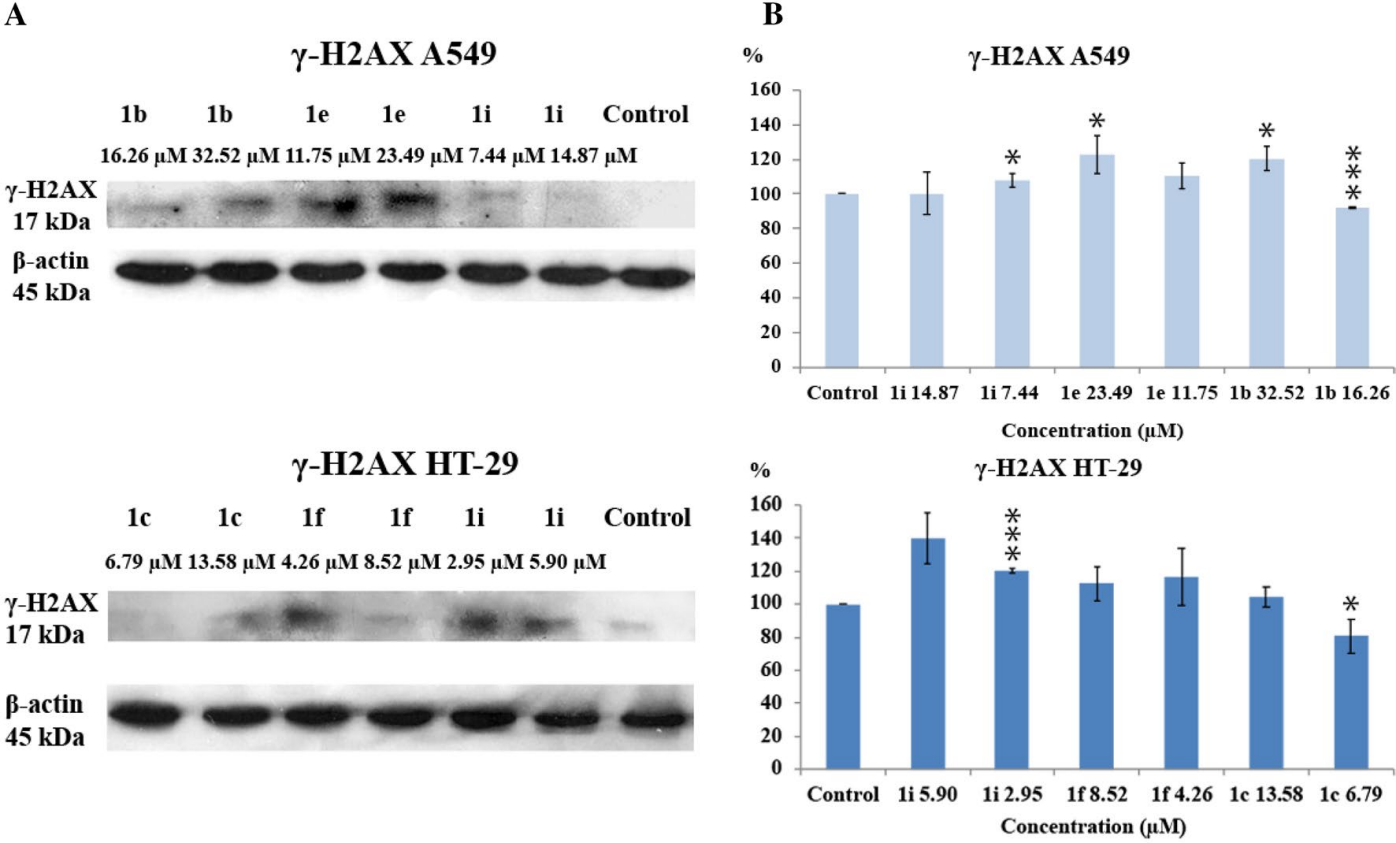
**a** Western blot analysis of γ-H2AX (17 kDa) protein levels in A549 and HT-29 cells after 24 h. Cells were treated with tetrahydroacridine derivatives at IC_50_ and _1/2_IC_50_ concentrations. **b** Relative densitometric values of γ-H2AX proteins levels. Protein quantification was performed by densitometric scanning. Experiment was repeated at least two times. Data were normalized using β-actin, indicated as mean ± SD and expressed as a percentage of control. One-way ANOVA analysis with post hoc analysis was performed. ***p < 0.001, *p < 0.05 were considered as significant in comparison to non-treated control

In order to test whether novel tetrahydroacridine could induce DNA damage which results in phosphorylate H2AX, A549 cells were treated with compounds **1b**, **1e**, **1i**, whereas HT-29 cells were treated with **1c**, **1f**, **1i** at their IC_50_ and _1/2_IC_50_ concentrations. In A549 cells, γ-H2AX were obtained in all samples after treatment with novel compounds. At IC_50_ concentration, the strongest effect was observed for **1e**, and slightly lower for **1b**. Compound **1i** had higher ability to damage DNA at _1/2_IC_50_ concentration (7.44 µM) than at IC_50_ concentration (14.87 µM) (Fig. 13). In HT-29 cells, the strongest H2AX phosphorylation was observed after treatment with **1i** and **1f**. Among compounds, **1c** had the lowest effect on DNA damage. The effect was noticed at IC_50_ (13.58 µM), but not at _1/2_IC_50_ (6.79 µM) (Fig. 13).

### In vitro study on cisplatin-sensitive and cisplatin-resistant 253J B-V cells

**Fig. 14:**
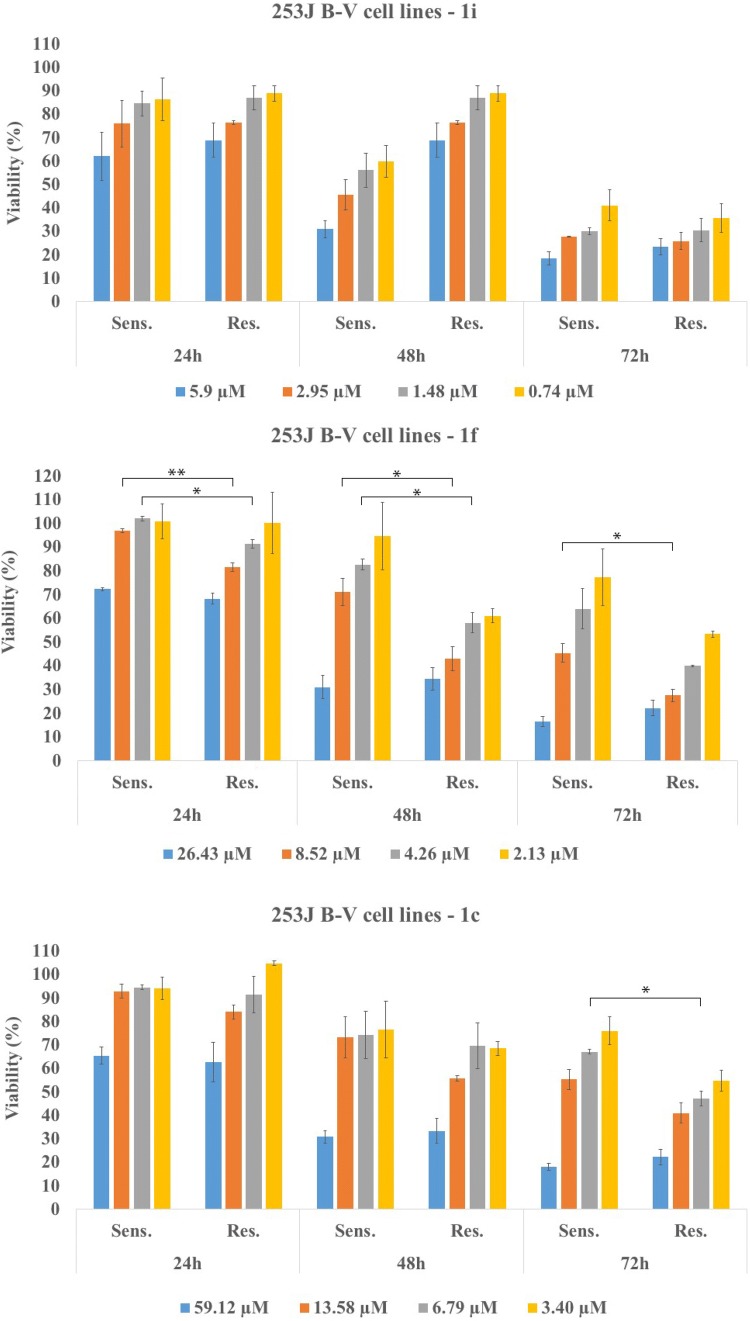

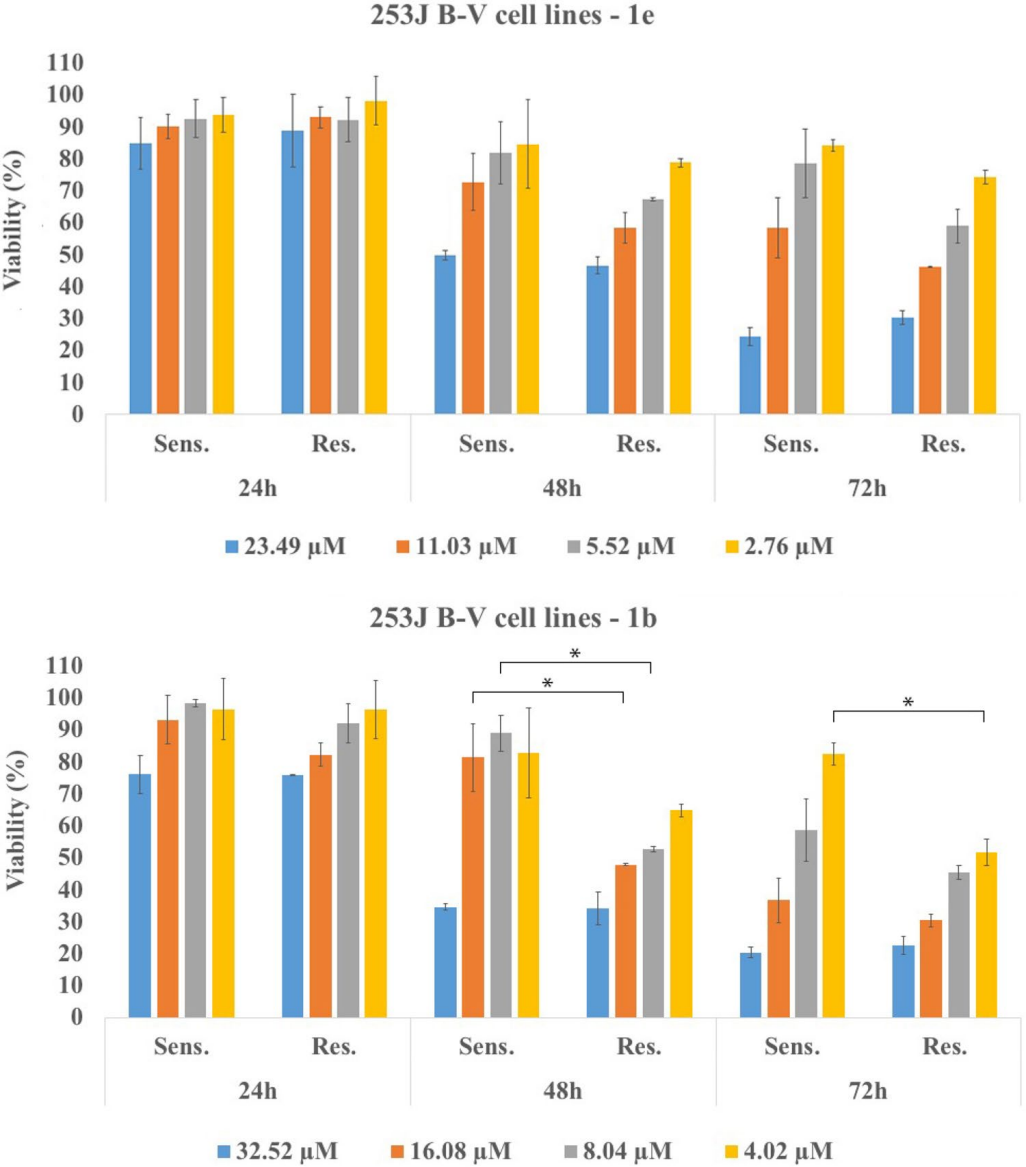
In vitro cytotoxic effect of tetrahydroacridine derivatives on cisplatin-resistant and non-resistant 253J B-V cells after 24 h, 48 h and 72 h of incubation. Experiments were done in quadruplicates and repeated two times. Statistical significance was assessed using one-way ANOVA analysis was performed. *p < 0.05, **p < 0.01 was considered as significantly different between viability of resistant and non-resistant cells

Since chemoresistance is a common feature during cancer progression, we would test the cytotoxic ability of our tetrahydroacridine derivatives with iodobenzoic acid moiety in cisplatin-resistant and non-resistant 253J B-V bladder cancer cells. 253J B-V cells were cultured in and exposed to the increasing concentration of cisplatin for 12 months. Resistance induction was described in publication by Ciamporcero et al. [36]. On the basis of MTT experiments in A549 and HT-29 cell lines, the compounds with longer linker (3 or 4 methylene groups) and highest cytotoxicity of each subgroup were chosen for this study. Compounds **1b**, **1c**, **1e**, **1f** and **1i** were tested in the various concentrations (IC_50_ of A549, IC_50_ of HT-29, _1/2_IC_50_, _1/4_IC_50_). Effect was measured after 24 h, 48 h and 72 h of incubation with compounds (Fig. 14). For sensitive and resistant cell lines, a similar cytotoxic effect was observed for each compound. The cytotoxicity increased over the time. One-way ANOVA analysis demonstrated that there were no significant differences of cytotoxicity between sensitive and resistant cells for most of the concentrations of compounds. However, for **1b**, **1c, 1f** differences were noticed after 24 h, 48 h or 72 h of incubation. It turned out, that resistant cells were more sensitive to the compounds **1b** (after 48 h at the concentrations of 16.08 and 8.04 µM; 72 h at the concentration of 4.02 µM), **1c** (after 72 h at the concentration of 6.79 µM), and to the **1f** (after 24 h and 48 h at the concentrations of 8.52 and 4.26 µM; after 72 h at the concentration of 8.52 µM) than non-resistant cells (Fig. 14). Taken together these results demonstrated that compounds displayed a strong cytotoxic activity in both sensitive and cisplatin-resistant cell lines. Acting against chemoresistance was observed mostly for compound **1f**, where this effect was observed for two concentrations after 24 h, 48 h and 72 h of observation.

### In vitro study on hyaluronidase activity

Inflammation is a biological response to aggressive agents or stimuli (pathogens, damaged cells, mechanical injury), involving immune and local vascular systems. Inflammation is controlled by anti-inflammatory mediators such as cytokines, chemokines and cellular enzymes (among them—hyaluronidase). Hyaluronidase is an enzyme, which depolymerises hyaluronan (part of extracellular matrix), its activity is increased during inflammation therefore the integrity of tissue is weakened during the inflammatory process. Chronic inflammation is commonly associated with the development of several diseases, such as Alzheimer’s disease, rheumatoid arthritis or cancers. [40, 73–76].

In the present study, the hyaluronidase inhibitory activity of novel compounds was determined by spectrophotometric assay. The IC_50_ values of tested compounds and the positive control, heparin, are presented in Table 3. All compounds presented good inhibitory activity in the range of IC_50_ values between 191.17 µM and 383.73 µM. The iodine substitution in *para* position enhanced inhibitory activity, as well as shorter carbon linker. The best compound was **1g** with 191.17 µM IC_50_, when heparin—had 56.41 µM IC_50_. It can be concluded that tetrahydroacridine derivatives could possess anti-inflammatory activity.

**Table 3:** In vitro hyaluronidase inhibitory activity of novel compounds **1a**–**1i** and a positive control, heparin

Compound	HYAL IC_50_ ± SD (µM)
**1a**	233.62 ± 7.90***
**1b**	232.68 ± 4.87***
**1c**	243.54 ± 7.95***
**1d**	299.56 ± 6.30***
**1e**	230.58 ± 5.64***
**1f**	383.73 ± 10.18***
**1g**	191.17 ± 10.78***
**1h**	200.65 ± 8.36***
**1i**	233.64 ± 8.98***
Heparin	56.41 ± 0.78

All values are presented as the mean ± SD; IC_50_, 50% inhibition of enzyme activity. Statistical significance was assessed using one-way ANOVA

***p < 0.001 was considered as significantly different from control heparin

### Cytoprotection against oxidative stress

Oxidative stress derives from an imbalance between formation and neutralization of ROS. It causes excessive oxidative damage to DNA, proteins and further to cells and tissues. These changes induce inflammatory diseases, cardiovascular diseases, aging and cancers. Drugs, which could have additionally anti-inflammatory and antioxidant properties, are appreciated as anticancer agents [77, 78]. In our study, we checked whether novel compounds possesses antioxidant properties (due to the benzoic acid moiety), which at low concentration could reverse side effects of anticancer therapies.

**Fig. 15:**
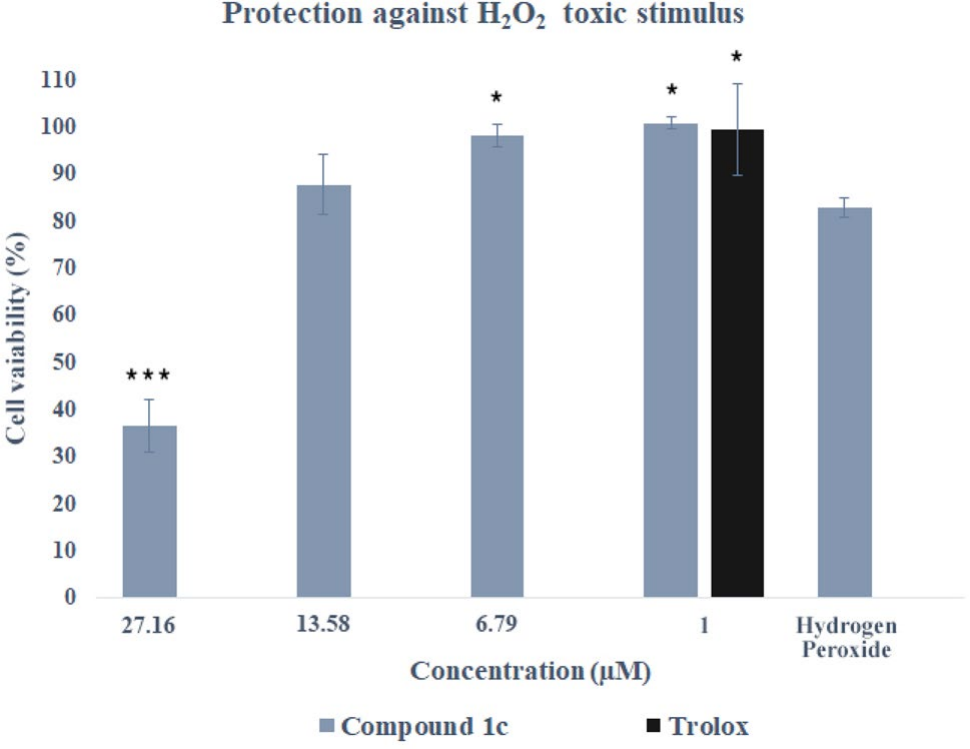
Protection by **1c** against H_2_O_2_ (100 µM) in SH-SY5Y cells. Cell viability was evaluated by MTT assay. Trolox was used as a reference antioxidant. Data were presented as the mean ± SD. One-way ANOVA analysis with post hoc analysis was performed. ***p < 0.001; *p < 0.05 was considered as significantly different comparing with H_2_O_2_-damaged cells without drug incubation

The cytoprotective properties of tetrahydroacridine derivatives against oxidative stress were measured in three toxicity models. In the first model, H_2_O_2_ was used to generate the exogenous free radicals. SH-SY5Y human neuroblastoma cells were incubated with compounds 24 h before addition of H_2_O_2_. After this time, toxic (100 µM) was added and maintained for the next 24 h in the presence of compounds. Five compounds were chosen (**1b**, **1c**, **1e**, **1f**, **1i**) and tested in four concentration: **1b**, at the concentrations of 65.04 µM, 32.52 µM (IC_50_ of A549), 16.26 µM and 1 µM; **1c**, at the concentrations of 27.16 µM, 13.58 µM (IC_50_ of HT-29), 6.79 µM and 1 µM; **1e**, at the concentrations of 46.98 µM, 23.49 µM (IC_50_ of A549), 11.75 µM and 1 µM; **1f** at the concentrations of 17.04 µM, 8.52 µM (IC_50_ of HT-29), 4.26 µM and 1 µM; **1i** at the concentrations of 29.74 µM, 14.87 µM (IC_50_ of A549), 5.90 µM (IC_50_ of HT-29) and 1 µM. Cell treated with H_2_O_2_ only (in the absence of novel compounds), showed a viability of 82.74% in comparison to non-treated control cells. The antioxidant trolox (6-hydroxy-2,5,7,8-tetramethylchroman-2-carboxylic acid), at the concentration of 1 µM, was used as positive control. Cells incubated with trolox showed viability of 99.38% and cytoprotection of 96.38%. Only compound **1c** provided small cytotoxicity (Fig. 15) and had cytoprotective property at concentrations of 13.58 µM, 6.79 µM—28.45%, 89.47%, respectively (Table 4). Rest of the compounds, apart from **1i**, had cytoprotection properties only at the concentration of 1 µM (**1b**, **1e**, **1f**—98.40%, 40.67%, 95.35%, respectively), which was even higher or slightly lower (for **1e**) than that of trolox. Almost all results were statistically significant in comparison to H_2_O_2_ treated cells (without any other compound) (Table 4). Incubation of **1i** with toxic stimulus resulted in high increase of cytotoxicity, therefore neuroprotection was not observed at any dose.

**Table 4:** Cytoprotection (%) in the cell line SH-SY5Y against H_2_O_2_ (100 µM) at the chosen concentrations

Cells viability (%) ± SD after 24 h of incubation with compound and H_2_O_2_				
Compound	Concentration (µM)			
**1b**	**65.04**	**32.52**	**16.26**	**1**
	17.21 ± 1.18***	24.93 ± 3.23***	64.61 ± 4.88**	99.20 ± 1.67**
**1c**	**27.16**	**13.58**	**6.79**	**1**
	36.66 ± 5.57**	87.65 ± 6.40	98.18 ± 2.49*	100.83 ± 1.19*
**1e**	**46.98**	**23.49**	**11.75**	**1**
	17.39 ± 2.15***	21.78 ± 3.21***	59.16 ± 6.70*	89.76 ± 8.55
**1f**	**17.04**	**8.52**	**4.26**	**1**
	23.85 ± 2.08***	36.99 ± 5.12***	79.45 ± 9.87	94.34 ± 7.26
**1i**	**29.74**	**14.87**	**5.90**	**1**
	18.74 ± 1.25***	20.72 ± 1.45***	25.95 ± 1.24***	29.68 ± 3.39***
Trolox				**1**
				99.38 ± 9.81*

One-way ANOVA analysis with post hoc analysis was performed

***p < 0.001; **p < 0.01; *p < 0.05 was considered as significantly different comparing with H_2_O_2_-damaged cells without drug incubation

Rotenone with oligomycin A (R/O) were taken to induce mitochondrial ROS by blocking of complexes I and V of the mitochondrial electron transport chain. It causes free radical production and blockade of ATP synthesis [79]. In the pre-incubation experiment, it could be assessed whether the compounds have cytoprotective properties due to the activation of endogenous antioxidant pathways. In the co-incubation could be shown whether compounds are free-radical scavengers [42, 80–82]. MTT assay was a test of choice, because in this assay mitochondrial activity is measured. Live cells can reduce MTT, whereas apoptotic or necrotic cells do not carry out this chemical modification. It can be determined whether novel compound would protect cells from both apoptotic and necrotic death resulting from the increase of intracellular ROS. In both experiments, trolox was used as a reference compound. In the pre-incubation experiment, SH-SY5Y cells were incubated with the compounds for 24 h before the addition of R/O mixture. After this time, toxic mixture was added, and cells were maintained for an additional 24 h in the presence of the compounds.

**Fig. 16:**
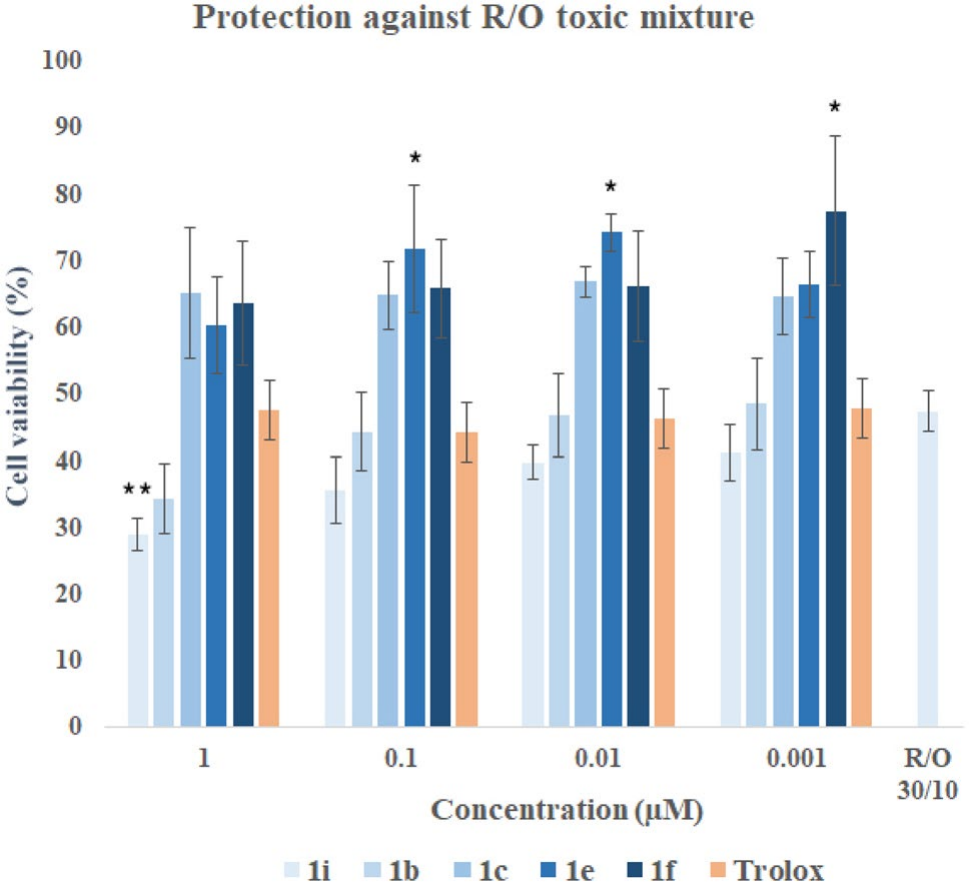
SH-SY5Y cells viability (%) of pre-incubation assay against mixture of rotenone (30 µM) and oligomycin A (10 µM) at the chosen concentrations of compounds **1i**, **1b**, **1c**, **1e**, **1f**. Trolox was used as a reference antioxidant. Data were presented as the mean ± SD. One-way ANOVA analysis with post hoc analysis was performed. **p < 0.01, *p < 0.05 was considered as significantly different comparing with R/O cells without drug incubation

In the pre-incubation assay, SH-SY5Y cells were treated with compounds (**1b**, **1c**, **1e**, **1f**, **1i**) in the range of concentration 1–0.001 µM. Cells exposed to the R/O mixture showed viability of 47.39% in the incubation without any compound (Table 5). Compounds **1b** and **1i** did not show cytoprotective properties at the chosen concentrations. Compound **1c**, **1e**, **1f** showed their action against oxidative stress in the whole range of concentrations (Fig. 16, Table 6) and were stronger antioxidants than trolox.

**Table 5:** SH-SY5Y cells viability (%) with ± SD of pre-incubation and co-incubation assays against mixture of rotenone (30 µM) and oligomycin A (10 µM) at the chosen concentrations

Compound	Concentration	Cell viability (%)			
		1 µM	0.1 µM	0.01 µM	0.001 µM
**1b**	Pre-incubation	34.41 ± 5.19	44.39 ± 5.77	46.92 ± 6.20	48.50 ± 6.87
	Co-incubation	37.28 ± 3.50	41.01 ± 6.63	42.54 ± 4.14	45.82 ± 4.69
**1c**	Pre-incubation	65.23 ± 9.74	64.83 ± 5.19	66.88 ± 2.33	64.71 ± 5.67
	Co-incubation	39.88 ± 5.68	39.09 ± 5.40	42.34 ± 5.08	44.86 ± 4.82
**1e**	Pre-incubation	60.43 ± 7.22	71.80 ± 9.55*	74.32 ± 2.80*	66.45 ± 5.06
	Co-incubation	35.62 ± 1.66*	37.61 ± 3.15	39.82 ± 2.24	43.60 ± 3.44
**1f**	Pre-incubation	63.60 ± 9.26	65.92 ± 7.34	66.15 ± 8.29	77.46 ± 11.20*
	Co-incubation	36.35 ± 2.34**	37.60 ± 2.39**	39.53 ± 1.11*	40.19 ± 0.42
**1i**	Pre-incubation	28.96 ± 2.35**	35.63 ± 4.86	39.82 ± 2.63	41.26 ± 4.21
	Co-incubation	33.69 ± 2.87*	34.51 ± 3.36*	39.92 ± 2.43	47.53 ± 2.30
Trolox	Pre-incubation	47.67 ± 2.46	44.26 ± 3.73	46.38 ± 2.06	47.91 ± 4.48
	Co-incubation	49.23 ± 8.49	48.09 ± 8.01	50.34 ± 8.11	50.83 ± 6.98

One-way ANOVA analysis with post-hoc analysis was performed

**p < 0.01, *p < 0.05 was considered as significantly different comparing with R/O cells without drug incubation

**Table 6:** Cytoprotection (%) of the compounds **1c**, **1e**, **1f** and trolox in the SH-SY5Y cells in the pre-incubation assay with mixture of rotenone (30 µM) and oligomycin A (10 µM)

Compound	Concentration			
	Cytoprotection			
	1 µM (%)	0.1 µM (%)	0.01 µM	0.001 µM
**1c**	33.92	33.15	37.05	32.91
**1e**	24.79	46.39	51.19	36.22
**1f**	30.81	35.21	35.65	57.16
Trolox	0.53	–	–	0.98

**Fig. 17:**
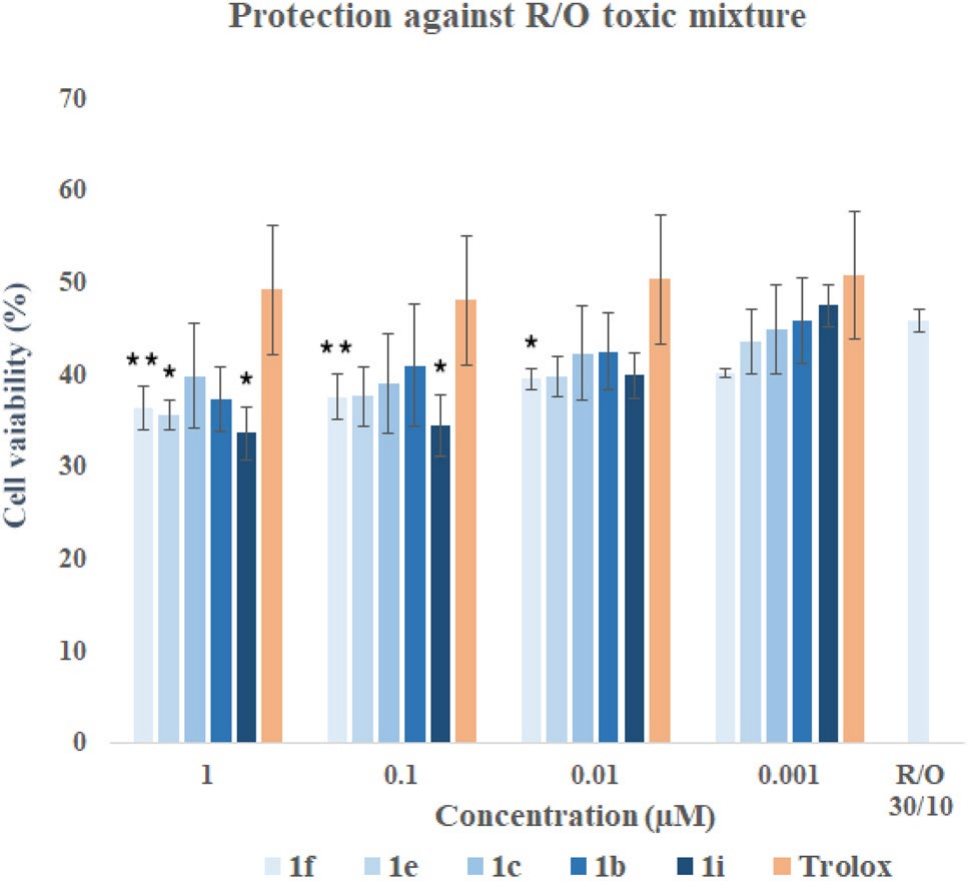
SH-SY5Y cells viability (%) of co-incubation assay against mixture of rotenone (30 µM) and oligomycin A (10 µM) at the chosen concentrations of compounds **1f**, **1e**, **1c**, **1b**, **1i**. Data were presented as the mean ± SD. One-way ANOVA analysis with post hoc analysis was performed. **p < 0.01, *p < 0.05 was considered as significantly different comparing with R/O cells without drug incubation

In the co-incubation assay, compounds and mixture of R/O were incubated together for 24 h. Cells exposed to the R/O mixture showed viability of 45.92% in the incubation without any compound. Trolox showed cytoprotective properties in whole range of concentration (1 µM, 0.1 µM, 0.01 µM, 0.001 µM—6.12%, 4.02%, 8.18%, 9.08%, respectively). It turned out that none of the compounds (apart from **1i** at the concentration of 0.001 µM and cytoprotection of 2.99%) had cytoprotective properties in the whole range of concentrations (Fig. 17). Compounds were not able to capture free radicals; therefore they cannot be classified as free-radical scavengers.

Moreover, we verified whether novel compounds could protect A549 and HT-29 cells against stress induced by H_2_O_2_. Cellular ROS was detected by measuring DCF fluorescence intensity. A549 and HT-29 cells were treated with compounds at IC_50_ concentration. Previous study on SH-SY5Y cell line confirmed, that compounds did not protect cells against H_2_O_2_ toxic stimulus, even if they were pre-incubated with compounds. Only **1c** showed cytoprotection against H_2_O_2_. ROS level after treatment only with toxic stimulus was increased 1.86- and 1.88-fold (for A549 and HT-29 cells, respectively) in comparison with non-treated control. Even higher ROS level than after treatment only with H_2_O_2_ was observed for A549 cells after incubation compounds. For **1i**, **1e**, **1b**—it was 1.98-fold increase in ROS level production. In HT-29 cells, compounds **1c**, **1f**, **1i** caused 1.83-fold, 1.87-fold and 1.85-fold increase in ROS level. It was only slightly lower than for H_2_O_2_ treatment. Results showed that compounds did not act again oxidative stress in A549 and HT-29 cells. Since cancer cells produce high level of ROS (which makes cancer cells more vulnerable to the agents that further increase ROS levels), the lack of ROS scavenger activity of novel compounds may represent an important clue to their use as chemotherapeutic drugs.

## Discussion

Cancerous diseases are the third reason of death worldwide. Among all cases, the most common cause of death in both sexes is lung cancer, when colorectal cancer is the third cause of death among other types of cancers [1, 2]. Among different groups of drugs used in chemotherapy, acridine derivatives, which are tricyclic heterocyclic compounds, have been extensively explored as potential therapeutic agents for the cancer treatment [83]. A basic mechanism of acridines is intercalation of DNA in space between base pairs due to plate, polycyclic aromatic structure. Overall, acridine derivatives have a long history in therapeutics. They are used as antibacterial agents (ethacridine lactate—rivanol) [84] or antimalarial agents (mepacrine) [85]. Luckily, they use in chemotherapy was also confirmed as the drug like amsacrine [86]. In order to obtain new derivatives of acridine, the amino group is modified, and different moieties are added to the structure. These processes lead to modify efficacy and toxicity of compounds. Novel compounds should damage DNA, induce apoptosis or arrest cell cycle in cancer cells. Induction of these processes are an important factors in effective chemotherapy.

We have synthesized and evaluated novel tetrahydroacridine derivatives with iodobenzoic moiety. Nine compounds were divided into three groups and tested against cancer cell lines A549 and HT-29 cells, and non-cancer cell line, EA.hy926 cells. The overall results showed that all compounds displayed high cytotoxicity activities towards cancer cell lines and were more effective than reference compounds. Moreover, HT-29 cells were more sensitive to tetrahydroacridine derivatives than A549 cells.

Reference compounds were chosen with justification. Recommended regimens for lung cancer include etoposide, cisplatin, paclitaxel or vincristine [87]. Moreover, many researchers in their work on A549 cell line, use etoposide as a positive control [12, 88]. Therefore, it was our justification to use etoposide as a reference compound in study. As a control for HT-29 cells, 5-fluorouracil was used. 5-fluorouracil is widely used in the treatment of various cancers, such as colorectal, breast, head and neck cancers. Among mentioned cancers, in colorectal cancer that 5-FU has the greatest impact. Against colorectal cancer, 5-FU is also used with a combination with other drugs as—FOLFOX (5-FU, leucovorin and oxaliplatin), CapeOx (capecitabine and oxaliplatin) or FOLFIRI (5-FU, leucovorin and irinotecan) [89–91]. Also many researchers in their work on HT-29 cell line, use 5-FU as a positive control. All compounds were tested for 24-h incubation, therefore also positive controls—etoposide and 5-fluorouracil were incubated with cells for 1 day. As it is seen, 5-fluorouracil has very small cytotoxic effect on HT-29 cells after 24-h of incubation, which was also confirmed in different publications. The highest cytotoxic effect on HT-29 cells starts after 5 days of incubation [92, 93]. Also 24 h of incubation A549 cells with etoposide provides average cytotoxic effect, which increases with time of incubation. After 72-h incubation with etoposide, the IC_50_ is in the range of 50 µM [94]–106 µM [95].

Structure–activity relationship presented that the position of iodine in the *para* position and longer linker (3–4 carbon atoms) best improved the cytotoxic effect. Among all derivatives, **1i** (iodine in the para position and 4 carbon atoms linker) was the most toxic towards both cell lines. Effect of cytotoxicity on EA.hy926 cells (human umbilical vein cells) is interesting issue in chemotherapy. Chemotherapy has many side effects. Not only cancer cells are killed, but also in normal cells apoptosis is induced. Anticancer agents showed their cytotoxic effects towards various non-cancer cells. Especially the endothelial damage is observed, which leads to many consequences. The damage can be classified into groups on the basis on mechanisms—direct endothelial damage, activation of coagulation factors, autonomic dysfunction, stimulation of fibroblasts. Damage can further lead to many side effects such as Raynaud’s phenomenon, cerebrovascular attacks, hypertension, myocardial ischaemia and infarction, pulmonary or hepatic veno-occlusive disease. Moreover, evaluation of endothelial function may be important to identify risk for cardiovascular events in patients who receive chemotherapy [96]. Etoposide, which is used in the treatment of lung cancer, induced apoptosis of non-cancer cells such as epithelial cells and thymocytes [97, 98]. Among agents, which also induce toxicity in endothelial cells, are doxorubicin, paclitaxel and bleomycin. The side effects in vivo with clinical impairments regards pulmonary fibrosis (by bleomycin), cardiovascular alteration (by doxorubicin), renal dysfunction were caused by injury of microvascularization. Etoposide and paclitaxel are less toxic towards endothelial cells than bleomycin and doxorubicin. All agents trigger apoptosis in human umbilical vein endothelial cells by Bcl-2, but not p53 [99]. On the other hand, paclitaxel, doxorubicin, etoposide can have an impact on anti-angiogenic or anti-vascular effects in vivo by inducing apoptosis of the endothelial cells surrounding the tumour (at the concentrations less toxic for the cells of the other veins and for cancer cells). However, bleomycin at its concentration triggers apoptosis of endothelial cells close to the level that inducing overall endothelium toxicity. Even though, all agents are used in chemotherapy. Moreover, better understanding of the mechanism endothelial injury caused by chemotherapy, might lead to better prevention from the damage [96, 100–102]. The results on non-cancer cell line suggested that selected compounds were more toxic towards HT-29 colorectal cancer cells than normal cells—EA.hy926 cell line. When compare results from EA.hy926 cells and A549 cells cytotoxicity, higher toxic effect was observed for endothelial cells. Similar effect was observed for etoposide, which was much more toxic on EA.hy926 cells than on A549 cells. Even though, etoposide and other anticancer agents, which are harmful for endothelial cells, are used in chemotherapy. But yet, novel tetrahydroacridine with iodobenzoic acid would be safer for distributing in veins and using towards colorectal cancer cells (HT-29) than lung cancer cells (A549). Moreover, ADMET prediction showed that almost all compounds did not violate Lipinski’s rule, had good pharmacokinetic profiles and would have good oral bioavailability. However, small problems of oral absorption might be observed for compounds **1e**–**1i.**

Multidrug resistance appears in tumour cells during administration of chemotherapy. It is a major problem during treatment, when cancer cells do not react to many drugs, such as etoposide, cisplatin, 5-FU. Therefore, successful treatment cannot be obtained and many patients die. Drug resistance can be attributed to various mechanisms, such as alterations in apoptotic pathways, enhanced expression of multidrug resistance-associated proteins, altered drug metabolism or uptake and overexpression of cytoprotective genes [103]. Taken into consideration drug resistance problem, preliminary test was performed. We tested the cytotoxic ability of our tetrahydroacridine derivatives with iodobenzoic acid moiety in cisplatin-resistant and non-resistant 253J B-V bladder cancer cells. We obtained similar (without statistical difference) cytotoxic effect for sensitive and resistant cell lines for compounds **1e** and **1i**. For **1b**, **1c**, **1f** difference were noticed after 24 h, 48 h or 72 h of incubation, where higher cytotoxicity appears in resistant cells than in non-resistant. However, for **1b** and **1c** stronger cytotoxic effect in resistant cells was observed only in few concentration and was not repeated in every time of incubation. Therefore, taken together these results demonstrated that almost all compounds displayed a strong cytotoxic activity in both sensitive and cisplatin-resistant cell lines. Acting against chemoresistance was observed mostly for compound **1f**, where higher cytotoxic effect in resistant cells was observed for two concentrations after 24 h, 48 h and 72 h of observation. Compound **1f** will be further examined on etoposide-resistant A549 cells and 5-fluorouracil-resistant HT-29 cells.

Inhibition of A549 and HT-29 proliferation were due to the inhibition of the progression cell cycle from G0/G1 to S phase. An accumulation in G0/G1 phase after treatment with **1i** was observed in A549 cells, and after treatment of **1c** in HT-29 cells. The nature of apoptosis and overall apoptosis was also determined. After treatment with compounds, late stage of apoptotic cells was observed in great amount and **1i** showed the highest level of total apoptosis (71.98% of apoptotic cells) at its highest concentration. Diverse effect was determined in HT-29 cells: greater part of total apoptosis consists of early apoptotic cells. Again, compound **1i** (at its highest concentration) caused the strongest apoptotic effect (24.10%), though it was significantly lower than one observed in A549 cells. In the colony forming assay, the highest cytotoxic effect (the smallest number of colonies) at the concentration of IC_50_ was observed for **1i** in A549 cells, and for **1c** in HT-29 cells. These results were comparable with results at IC_50_ from cell cycle arrest assays, where **1i** at caused the strongest inhibition of cell cycle in A549, and **1c** in HT-29 cells. Similar effects were observed for comparison of colony forming and apoptosis results. The strongest apoptotic effect and the lack of colonies were observed for 2xIC_50_ concentration of all compounds, beside **1b**. The results were comparable for apoptosis and colony forming at the IC_50_ concentration. At the IC_50_ concentration, the highest apoptotic effect and the smallest number of colonies were observed for **1i** in A549 cells, and for **1c** in HT-29 cells. Caspases activation is one of the most important signalling pathways that is involved in apoptosis—caspases play the key role in the initiation and execution of apoptosis. Caspases cleave PARP-1 (which is a substrate for caspases) between Asp214 and Gly215 residues in order to produce 89 kDa and 24 kDa fragments. PARP-1 cleavage (observed as 89 kDa) confirmed that all compounds activated apoptotic pathway due to the caspases activation, because PARP-1 is a substrate for caspases 3 and 7. Therefore to test apoptosis pathway, we used PARP-1 in our assay. The PARP-1 cleavage in A549 cells was more evident than in HT-29 cells. Considering IC_50_ concentrations, **1e** and **1i** caused the highest PARP-1 cleavage for A549 cells, whereas for HT-29 cells the strongest cleavage was observed for **1i** at the IC_50_ concentration. DNA damage was confirmed in H2AX phosphorylation analysis. Consistent results—induction of G1 cell cycle arrest and H2AX phosphorylation—were obtained for another group of tetrahydroacridine derivatives with fluorobenzoic moieties [12, 13]. DNA damage targets two cell cycle checkpoints—G1/S and G2/M. DNA damage induces program that blocks cells at one of these checkpoints until damage is repaired or cells are turn into apoptosis [104].

We checked whether addition of benzoic acid derivatives to the tetrahydroacridine influence on antioxidant activities [32]. Except for three interferences (tangeretin with tamoxifen, NAC with doxorubicin and beta-caroten with 5-fluorouracil), many data show the increase in effectiveness as well as decrease of side effects of chemotherapeutic drugs when administrated with antioxidants [25]. We performed a few studies on anti-inflammatory and antioxidant activities of novel compound. The inhibition of hyaluronidase activity suggested that compounds possess small anti-inflammatory properties. Cytoprotection against mitochondrial ROS was not observed in the co-incubation assay. On the contrary, it turned out that longer cell incubation with low doses of drugs (lower than IC_50_) might induce endogenous antioxidant pathways after treatment with **1c**, **1e**, **1f**, so these compounds had little cytoprotection activity, and only at doses lower than IC_50_. Compounds were not able to capture free radicals; therefore they cannot be classified as free-radical scavengers. In the last study, IC_50_ concentration of the drugs lead to increase of ROS production in A549 and HT-29 cells, which can relate to induction of apoptosis in cancer cells. To conclude, three compounds at the concentrations lower than IC_50_ had small cytoprotection activity (which can relate to the possibility of decrease of side effects in chemotherapy), and at IC_50_ concentrations all compounds had no protection activity, which may further result in the death of cancer cells. To conclude, iodobenzoic acid has almost no impact on antioxidant activity, because antioxidant effect was observed at low level.

In summary, among all compounds, **1c** and **1i** stood out. Both compounds have 4-carbons linker, which may contribute to cytotoxic activity. Even though higher cytotoxicity in MTT assay was observed for HT-29 cells than for A549, stronger cell cycle arrest, apoptosis, PARP-1 cleavage and DNA damage was confirmed for A549 cells. Further study will be carried out to determine another protein signalling of cell death and to explain their influence on combating of chemoresistance.
